# Agreement between patients’ and radiation oncologists’ cancer diagnosis and prognosis perceptions: A cross sectional study in Japan

**DOI:** 10.1371/journal.pone.0198437

**Published:** 2018-06-08

**Authors:** Lisa Jane Mackenzie, Mariko Leanne Carey, Eiji Suzuki, Robert William Sanson-Fisher, Hiromi Asada, Masakazu Ogura, Catherine D’Este, Michio Yoshimura, Masakazu Toi

**Affiliations:** 1 School of Medicine and Public Health, The University of Newcastle, Callaghan, New South Wales, Australia; 2 Graduate School of Medicine, Kyoto University Hospital, Kyoto, Japan; 3 Breast Surgery, Kyoto University Hospital, Kyoto, Japan; 4 Department of Nursing, Kyoto University Hospital, Kyoto, Japan; 5 Department of Radiation Oncology and Image Applied Therapy, Kyoto University Hospital, Kyoto, Japan; 6 National Centre for Epidemiology and Population Health, Research School of Population Health, Australian National University, Acton, Australian Capital Territory, Australia; Weill Cornell Medical College in Qatar, QATAR

## Abstract

This study assessed agreement between radiation oncologist- and cancer patient-reported perceptions about cancer diagnosis, time since diagnosis, treatment purpose, and whether life expectancy had been discussed; and described preferences for prognosis discussions. Adult cancer patients receiving radiotherapy at a Japanese hospital were invited to complete a touchscreen tablet survey. Patient survey responses were linked and comparisons made with a survey completed by their radiation oncologist. Among 146 cancer patient-oncologist dyads, there was almost perfect agreement on cancer diagnosis (ĸ = 0.88, 95% CI: 0.82–0.94), substantial agreement on time since diagnosis (ĸ = 0.70, 95% CI: 0.57–0.83) and moderate agreement on whether treatment goal was curative or palliative (ĸ = 0.44, 95% CI: 0.28–0.57; all *p*’s < 0.0001). Agreement about whether a life expectancy discussion had occurred was less than expected by chance (κ = -0.06, *p* = 0.9). Radiation oncologists reported that they had spoken to over two thirds of patients about this, whilst less than one third of patients stated that this discussion had occurred with their radiation oncologist. Over half of the patients who had not discussed life expectancy wanted to. Patients had variable preferences for whether they (80%), their radiation oncologist (78%) or their partner/family (52%) should decide whether they discuss their life expectancy. Although patient self-reported information about diagnosis and time since diagnosis appears to be reasonably accurate (compared with clinician-reported information), limitations of self-reported data about prognostic discussions were highlighted by poor agreement between patient- and clinician-reported information about whether prognostic discussions have occurred. Additional support is needed to improve prognosis communication and understanding in radiation oncology settings.

## Introduction

Cancer is a leading cause of death and morbidity worldwide [1]. For patients, having an accurate understanding of their diagnosis, prognosis and goals of treatment can facilitate informed treatment decision making [2] and can influence important patient-centred outcomes such as reduction in aggressive end-of-life medical care and improvements in quality of life [3, 4]. The provision of information about diagnosis and prognosis is important to most cancer patients in Western countries [5, 6], and oncologist communication of this key information is increasingly considered to be part of standardised cancer care [7, 8]. However, patient misunderstanding of their illness may vary due to limited education, denial, poor interpersonal communication [9, 10], or as a result of family and health professional information "gate keeping" [10–12]. Few studies have examined agreement between oncologist-reports and cancer patients’ understanding of their diagnosis [13], purpose of treatment [14], preferences for and perceptions of life expectancy discussions [15–17].

Cancer patients’ preferences for prognostic information are complex, and may vary across individuals and cultures. While up to 81% of cancer patients in Western countries want to discuss their life expectancy [5, 6], it has been reported that a smaller proportion of patients in Japan want this information [18]. Western guidelines recommend a patient-centred approach that is responsive to individual preferences for if, when and how much information about prognosis is discussed [19–21]. A more paternalistic approach to diagnosis and prognosis disclosure, where their doctor and/or family decides if and how much patients are told, may be more common in non-Western cultures [22–24]. Recent research with Chinese cancer patients and Australian Asian migrants suggests that this disclosure method may be more reflective of family and clinician preferences, rather than the preferences of patients [25, 26]. A study with 201 patients and 40 physicians in Japan more than two decades ago identified that clinicians typically underestimated the level of information that patients wanted [13].

Radiotherapy is a major treatment modality for cancer [27], with utilisation rates of between 48–52% recommended in Western settings [27]. Rates of utilisation have been increasing in Japan over the past two decades, from approximately 15% of all newly diagnosed cancer cases being treated in 1990, to approximately 28% during in 2009 [28]. Reports from Western settings indicate that it could be expected that the purpose of radiotherapy treatment be curative for 78–84% and palliative for 16–22% of cancer cases treated with radiotherapy [29]. Despite patient life expectancy often being overestimated by radiation oncologists [30, 31], it is reasonable to expect that treatment goals would have been discussed with patients when seeking informed consent for radiotherapy [2, 19]. Research into cancer patients’ prognosis disclosure experiences has largely focused on patients diagnosed with advanced cancer or specific tumour types [32–35]. This study addresses a need to explore prognosis disclosure experiences and understanding among a heterogeneous sample of early- and advanced-stage cancer patients with a range of diagnoses and prognoses [36]. The objectives of this study were to: Assess agreement between radiation oncologist- and patient-reported information about the patient’s cancer diagnosis, time since diagnosis, purpose of treatment, whether prognosis (life expectancy) had been discussed; describe the proportion of cancer patients who have not discussed life expectancy with their radiation oncologist, but want to; and describe radiation oncologists’ and patients’ views about who should decide whether life expectancy is discussed.

## Materials and methods

### Ethics approvals

Approvals were obtained from the University of Newcastle Human Research Ethics Committee (reference H-2011-0310) and Kyoto University Hospital Institutional Review Board (reference E1324), and investigations were conducted according to the principles expressed in the Declaration of Helsinki.

### Study design

Cross-sectional survey of patients and radiation oncologists.

### Sample and setting

Participants were recruited from a radiotherapy (RT) department in a large University hospital in Japan between April and July 2012. Patients who were aged 20 years or more, receiving external beam radiotherapy for cancer, and were physically and mentally able to participate were eligible for the study. Patients who were unable to provide informed consent and complete the survey in Japanese were excluded, as were those with less than two weeks of their treatment course within the study period (to ensure adequate time for eligibility screening, introductions and recruitment).

### Procedure

Nursing staff assessed patients attending the RT treatment centre for eligibility, and provided them with a study summary flyer. Nursing staff introduced interested patients to the study research assistant (RA) who explained the study and sought informed consent. Patients who provided written informed consent to having their survey responses compared to their radiation oncologists’ survey responses were asked to write their name and date of birth on a paper slip alongside their unique study identification number. At the conclusion of each recruitment day, this form was attached to a radiation oncologist survey with the corresponding number by the RA and distributed to the relevant radiation oncologist/s. All participating radiation oncologists provided written informed consent.

### Measures

#### Patient questionnaire

The patient survey was administered via Acer Iconia Tab A500 using the Rollapoll application (CREOSO Corp, Phoenix, Arizona). Awareness of a cancer diagnosis was assessed with the question "Do you know your diagnosis?". Participants who knew their diagnosis were asked their primary cancer diagnosis (response options: "Breast"; "Colorectal"; "Prostate"; "Lung"; "Melanoma"; "Don’t know" and "Other (please specify)", diagnosis month and year, and “What do you understand to be the main aim of your current treatment?” (response options: "To cure the cancer"; "To prevent the cancer from coming back"; "To control symptoms of cancer [cure is not possible]"). Participants who were aware of their diagnosis were asked to indicate whether they were willing to answer survey questions about life expectancy [37]. Participants willing to complete this section were asked "Have you and your radiation oncologist talked about your life expectancy?" Those who answered "yes" to the previous question were asked single-choice closed-ended questions, including "How did the discussion about life expectancy begin?" (response options: “I asked my doctor if we could talk about it”; “My doctor asked me if I wanted to talk about it”; “My doctor discussed it without asking me first”; “Other”); and "While my radiation oncologist cannot be certain, he/she has suggested that currently…" (response options: “My cancer diagnosis will not affect my life expectancy”; “It is far too early to tell”; “I will live more than 5 years”; “I will live for 2–5 years”; “I will live for less than 2 years”). Respondents who indicated that they had not talked to their radiation oncologist about their life expectancy were asked "Would you like to talk to your doctor about your life expectancy?" (response options: “Yes”; “No”). Participants also indicated their level of agreement with five non-mutually exclusive statements relating to their preferences for who should decide whether they discuss life expectancy with their radiation oncologist. Questions and response options (see S1 File) were based on those used by Mackenzie *et al* [36].

#### Radiation oncologist questionnaire

A paper survey provided to radiation oncologists (see S2 and S3 Files for the English and Japanese versions, respectively) asked similar questions about patients’ disease characteristics, purpose of treatment, and life expectancy discussions.

### Statistical analysis

Patient and radiation oncologist surveys were matched by a unique identification code, and agreement was assessed by comparing patients’ and radiation oncologists’ responses to equivalent questions. If a radiation oncologist or patient response was missing, the pair was excluded from analysis for that item. Patient- and radiation oncologist-reported cancer diagnoses were recoded into the following diagnostic groups: breast, prostate, lung, oesophageal, head and neck, other, don’t know, or patient not aware of cancer diagnosis. If a patient or radiation oncologist reported a diagnosis of "other" cancer types, responses were checked by a breast cancer surgeon (ES) to ensure consistent English translation. Days since diagnosis was calculated based on participants’ survey completion date (using the 15^th^ of the month to approximate diagnosis dates), and then categorised as: i. < = 90 days; ii. >90 to < = 180 days; iii. >180 to < = 365 days; and iv. >365 days. Observed agreement and Cohen’s κ or bias adjusted weighted κ with 95% CIs (estimated using bootstrapping with 1000 repetitions) are reported. Extent of agreement was assessed using Cohen’s kappa estimates (qualitatively classified as: <0 = less than chance agreement; 0.01–0.20 = slight agreement; 0.21–0.40 = fair agreement; 0.41–0.60 = moderate agreement; 0.61–0.80 = substantial agreement; 0.81–0.99 = almost perfect agreement) [38]. *P*-values are reported for the hypothesis tests that kappa = 0 (i.e. agreement is not greater than chance), and a significance level of 5% used.

The percentage of patients who had not discussed their life expectancy with their radiation oncologists but wanted to was also estimated with 95% Confidence Intervals (CIs). The percentage of patients and radiation oncologists agreeing or strongly agreeing that life expectancy disclosure should be (non-mutually exclusively) patient-determined, partner/family determined, and clinician-determined was estimated with 95% CIs. Life expectancy disclosure responses mapped to these categories are described in S1 Table. Data analyses were conducted using Stata version 11.2 (Statacorp LP, College Station, TX) software.

### Sample size

An overall sample of 150 patients (with a 70% response rate to the optional life expectancy section) would allow prevalence estimates with 95% CIs within ±15% of the point estimate for kappa of 0.5 or higher (assuming proportions of 50%).

## Results

### Consent rates—Patient and radiation oncologist survey

Of 262 eligible patients, 152 completed surveys were eligible for inclusion in the analysis (completion rate 58%) [39]. Of the patients with completed surveys, 151 also gave consent to have their responses compared to their radiation oncologist’s. A total of 16 radiation oncologists participated in the study. Four radiation oncologist surveys were missing the unique ID number required for data linkage, and one radiation oncologist survey was not returned to the research team. This left a total of 146 patient-radiation oncologist survey pairs available for analysis. Fifty-five percent of patient respondents were male. Patients were a median of 64 years of age (Q1: 58, Q3: 72) and had a median of 12 years of education (Q1: 12, Q3: 16). The majority of patients (n = 120; 82%) reported that they lived with their husband, wife or partner.

### Agreement between patients and radiation oncologists about cancer diagnosis and treatment purpose

Table 1 presents data on patients’ cancer diagnosis categories, based on patient and radiation oncologist survey responses. All 141 (97%) patients who were aware of their diagnosis reported that they were informed of this by a doctor (not by family or others). There was 90% observed agreement between patient- and radiation oncologist-reported cancer diagnosis category, with a Cohen’s ĸ of 0.88 (95% CI: 0.82, 0.94, p < 0.0001) indicating almost perfect agreement. Of the 14 cases where there was disagreement between patient- and radiation oncologist-reported cancer type, five patients indicated that they were unaware they were diagnosed with cancer, and another five that they did not know what their cancer type was.

**Table 1:** Patient-reported and radiation oncologist-reported cancer diagnosis (n = 146).

	Prevalence (by data source)	
Cancer diagnosis	Patient report	Radiation oncologist report
	n (%)	n (%)
Breast	37 (25%)	38 (26%)
Prostate	37 (25%)	37 (25%)
Lung	14 (9.6%)	15 (10%)
Oesophageal	12 (8.2%)	11 (7.5%)
Head & Neck ^a^	10 (6.8%)	15 (10.2%)
Other ^b^	26 (18%)	30 (21%)
Don’t know	5 (3.4%)	0 (0%)
Not aware of cancer diagnosis	5 (3.4%)	N/A (0%)

^a.^ “Head & Neck Cancer” category includes the following responses: head and neck cancer, pharyngeal cancer, hypopharyngeal cancer, oropharyngeal cancer, nasal cavity cancer, nasopharynx cancer, paranasal cavity cancer, oral cavity cancer, maxillary sinus cancer, laryngeal cancer, tongue cancer, ethmoid sinus cancer, right maxillary gingival cancer

^b.^ “Other Cancer” category includes the following responses: kidney cancer, cholangiocarcinoma, pituitary tumor, pancreatic cancer, malignant lymphoma, sarcoma, brain tumor, benign meningioma, ascending colon cancer liver metastasis, sacro-iliac bone cancer, ovarian cancer, mesothelioma, tumor, cervical cancer, follicular lymphoma, gastric cancer, optic nerve meningioma or optic lymphoma, endometrial cancer, merkel cell cancer, anal canal cancer, intrahepatic bile duct cancer, liver cancer, malignant pleural mesothelioma, meningioma, pituitary adenoma, pleural mesothelioma, skin cancer, urachal cancer

Table 2 shows the numbers of patients and radiation oncologists who reported length of time since the patient’s cancer diagnosis within each category, and provides an indication of agreement between these two sources. Observed agreement between patients and radiation oncologists regarding the approximate number of months since cancer diagnosis was 91%, and a weighted ĸ of 0.70 (95% CI: 0.57, 0.83, p < 0.0001) indicating that agreement was substantial. There was greater than 12 months discrepancy between patient- and radiation oncologist-reported time since diagnosis for 17 survey pairs. The maximum discrepancy was 151 months.

**Table 2:** Number and percentage of patients who agreed with radiation oncologists on approximate length of time since cancer diagnosis (n = 137).

	Radiation oncologist report				
Patient report	90 days or less	91–180 days	181–365 days	365 days or more	TOTAL
90 days or less	**31 (79%)**	0 (0%)	3 (7.7%)	5 (14%)	39
91–180 days	3 (7.7%)	**21 (91%)**	3 (7.7%)	2 (5.6%)	29
181–365 days	3 (7.7%)	2 (8.7%)	**28 (72%)**	1 (2.8%)	34
365 days or more	2 (5.1%)	0 (0%)	5 (13%)	**28 (78%)**	35
**TOTAL**	39	23	39	36	137

Table 3 shows the number and percentage of patients who agreed and disagreed with their radiation oncologist regarding the purpose of treatment. Observed agreement between patients and radiation oncologists regarding the aim of treatment was 72%, with a Cohen’s ĸ of 0.44 (95% CI: 0.28, 0.57, p < 0.0001) indicating moderate agreement.

**Table 3:** Number and percentage of patients who agreed with their radiation oncologists about the aim of current cancer treatment (n = 138).

	Radiation oncologist report			
Patient report	To cure the cancer	To prevent the cancer coming back	To control symptoms (cure not possible)	TOTAL
To cure the cancer	**73 (86%)**	12 (36%)	12 (60%)	**97**
To prevent the cancer coming back	9 (11%)	**21 (64%)**	3 (15%)	**33**
To control symptoms (cure not possible)	3 (3.5%)	0 (0%)	**5 (25%)**	**8**
**TOTAL**	**85**	**33**	**20**	**138**

### Agreement between patients’ and radiation oncologists’ about whether life expectancy was discussed

Of 146 completed radiation oncologist surveys, radiation oncologists indicated that they had spoken to 67% of patients (n = 98) about how cancer might influence their life expectancy. Radiation oncologist responses indicated that cancer was not likely to affect the life expectancy of 14% of patients (n = 21), while 42% (n = 62) were expected to live for more than five years; 22% (n = 32) for 2–5 years and 21% (n = 31) less than two years.

Of the 141 patient respondents who indicated that they were aware of their cancer diagnosis, 82% (n = 116, 95% CI: 75%, 88%) were willing to answer questions about their life expectancy. Both patient and radiation oncologist responses to the question about whether a life expectancy discussion had occurred were available for 113 survey pairs. Table 4 shows the percentage of patients and radiation oncologists reporting that life expectancy had been discussed. Radiation oncologists reported that they had spoken to 79 patients (70%, 95% CI: 61–78%) about how cancer might influence their life expectancy. However, only 19 patients (17%, 95% CI: 10–25%) reported that they had discussed life expectancy with their radiation oncologist, and only seven could provide a specific life expectancy estimate. Over half (58%; 95% CI: 46–68%) of patients who reported that they had not discussed life expectancy wanted to. There was 33% observed agreement between patients and radiation oncologists on whether life expectancy had been discussed, with Cohen’s κ = -0.06 (95% CI: -0.17, 0.05, *p* = 0.89) indicating that agreement was less than what would have been expected by chance.

**Table 4:** Number and percentage of patients reporting life expectancy disclosure experiences that agreed with their radiation oncologist (n = 113).

	Radiation oncologist perceived disclosure		
Patient perceived disclosure	Yes	No	TOTAL
Yes	**11 (14%)**	8 (24%)	**19**
No	68 (86%)	**26 (76%)**	**94**
**TOTAL**	**79**	**34**	**113**

### Perceptions about who should decide whether life expectancy is discussed

**Fig 1:**
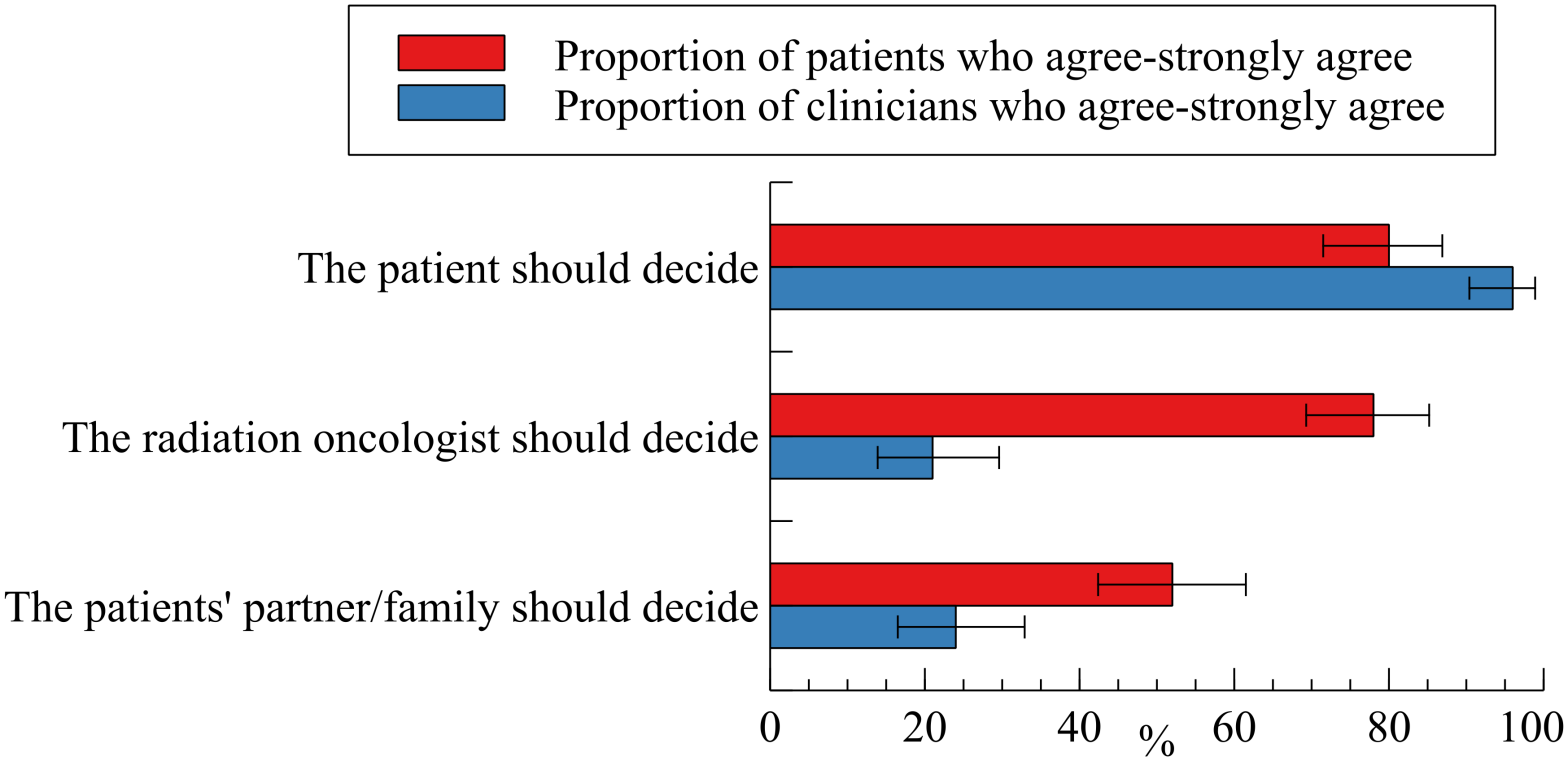
Percentage (and 95% CIs) of patients and radiation oncologists who agreed or strongly agreed with each (non-mutually exclusive) life expectancy disclosure approach preference.

Fig 1 shows the proportion of patients and radiation oncologists who agreed or strongly agreed to items indicating that the patient, radiation oncologist, and partner/family should decide when life expectancy information is disclosed. Patients had mixed preferences for the patient, radiation oncologist and partner/family to decide whether life expectancy is discussed. The majority of radiation oncologists agreed that the patient should decide whether they discuss how cancer may influence their life expectancy.

## Discussion

Few studies examine the concordance between radiation oncologists’ and cancer patients’ perceptions of key information related to the diagnosis: cancer type,[13] aims of treatment,[14] preferences for and perceptions of life expectancy discussions [15–17, 40, 41]. As such, this study provides guidance on areas of strengths and weaknesses in doctor-patient communication about these topics.

Agreement between cancer patients and their radiation oncologist regarding type of cancer diagnosis and months since diagnosis was found to be substantial to almost perfect. In 1992 an estimated 18% of cancer patients in Japan were informed of their diagnosis (based on reports of bereaved family carers),[42] while in our 2012 study 88% of patients were able to accurately report their diagnosis. Increased rates of cancer diagnosis awareness may reflect changes in hospital policies, increased emphasis on patient autonomy and patient centred care, and increased availability of communication skills training programs for cancer clinicians [43].

The radiation oncologist sample reported similar proportions of patients received treatment with palliative intent (14%) to what we would expect based on reports from Western settings (16–22%) [29]. However, sixty percent of patients whose radiation oncologist indicated they were treating with a palliative aim (i.e. reported the treatment aim was “to control symptoms [cure not possible]) believed they were being treated curatively. Agreement between patients and radiation oncologists on treatment aim ranged from fair to moderate, suggesting that patients understand less about their prognosis than about their diagnosis. This comparatively low agreement between patients and radiation oncologists may be due to physician factors such as inability to convey information about this issue in a way which is understandable to the patient [44] or concerns about the lack of adequate psychosocial support to help patients cope with knowledge of a poor prognosis [10]. There may also be patient factors at play, such as denial [2, 44].

The aforementioned finding that 60% of patients reported an overly optimistic treatment goal may be a result of their not having an accurate understanding of their life expectancy [10], with the majority of patients (83%) reporting that they had not discussed life expectancy with their radiation oncologist. This figure is higher than the 47% of radiotherapy patients who reported that they had not discussed life expectancy with their clinician in a recent Australian study [36], suggesting potential cultural or health care differences. An underlying cultural factor that may be influencing high rates of patient-reported non-disclosure of life expectancy estimates is physicians often having to navigate disclosure interactions within traditional family-centric consultation models [45, 46] whether or not there is concordance between patient and family disclosure preference on if and how this is done [35, 47]. Additionally, Japanese cultural norms of maintaining harmony and respect may lead to patient hesitation in asking questions and expressing information preferences within patient-physician interactions [45, 48].

Of the participants who reported they had not discussed life expectancy, 58% indicated that they wished to, consistent with the Japan-based work of Fujimori and colleagues reporting 50% of people with cancer would like to be told their life expectancy if they were to receive bad news [18]. Similar within-culture variation in individual patients’ preferences for disclosure of life expectancy information has been reported in Western settings [36, 49]. Taken together with the finding that radiation oncologists were able to provide a life expectancy estimate for all 146 patients (whilst only 19 patients indicated they had discussed this and only seven patients were able to report such an estimate), this unmet need suggests a requirement to better elicit and respond to patients’ preferences for life expectancy information in this setting. There are likely to be common factors across Western and non-Western settings that contribute patient-reported non-disclosure of life expectancy information, including time pressure within the oncology consultation [50], physician discomfort with discussing life expectancy [32, 51], as well as patient health literacy and recall of information [32]. Given that Japanese patients may be unlikely to ask questions or directly express their information preferences during medical consultations, physicians’ use of open-ended questions about information preferences may bring about improved patient satisfaction with cancer care communication [52].

Our finding of poor agreement between clinicians and patients regarding life expectancy disclosure and aims of treatment must be understood in the context of patient and clinician views about how such discussions should be initiated. Our data indicated substantial differences between clinician and patient views regarding who should determine whether a life expectancy discussion takes place. Radiation oncologists agreed that the patient should decide (96%); while a much smaller percentage agreed that the doctor should decide (21%); and the family should decide (24%) whether life expectancy is discussed. In contrast, 80% of patients indicated that they should decide themselves; 78% that the radiation oncologist should decide; and 52% that the family should decide whether life expectancy should be discussed. A survey of the general population in Japan (aged between 40–60 years) identified similar overlapping preferences for self, clinician and family directed disclosure of life expectancy information [53]. These complex patient preferences for shared decision-making have been previously reported in international settings [54, 55], and appear to be consistent with what might be expected in consensus-based family-centric medical communication and decision-making models in Japanese settings [56]. Our findings suggest that while radiation oncologists may be waiting for patients to take the initiative in raising the issue of life expectancy during treatment (either with them or another clinician responsible for cancer treatment);[44] patients may be expecting clinicians to take a more active role in starting the discussion prior to and during RT [57]. This may account, in part, for discrepancies between patient- and radiation oncologist-reported life expectancy disclosure experiences.

It is possible that generalisability of the current results to patients receiving radiotherapy may be limited due to recruitment from a single university hospital, and the opt-out life expectancy section (with no electronic response validation applied to allow respondents to easily skip questions within this section) which produced a smaller sample (82%) and some missing data. A limitation is that although we included a broad range of cancer types, our sample was not sufficient to enable us to explore whether there were significant differences between the experiences of people with different types of cancer. Radiation oncologist surveys were not identifiable, and we were not able to assess radiation oncologists’ sociodemographic or training characteristics or variations in preferences for disclosure and other outcomes. Additionally, we did not assess whether physicians had disclosed life expectancy information to family members. This study did not assess whether patients had discussed their life expectancy with another healthcare provider involved in their cancer care, with the study focus instead being on assessing whether treating radiation oncologists discuss radiotherapy treatment goals with patients when seeking informed consent for the treatment [2, 19]. Although this study was limited to patient and radiation oncologist self-reported life expectancy disclosure experience (rather than being supplemented with more objective measures such as audiotaped consultations), this allowed us to assess patients’ understanding taken from consultations.

### Research and practice implications

These findings should be reassuring for researchers in cancer treatment settings relying on patient self-reported information about diagnosis and time since diagnosis, with high agreements rates suggesting reasonable accuracy. However, differing views between patients and radiation oncologists about the purpose of treatment and whether a life expectancy discussion had occurred highlights the limitations of both patient- and clinician-reported information about whether prognostic discussions have occurred and what was communicated.

Given that many patients had not discussed life expectancy but wanted to, there is a need to address how these discussions are initiated in clinical practice. In Western settings, a patient-centred approach is recommended (i.e. the doctor should ask the patient if they want to discuss life expectancy). However, our findings also support past research that indicated that not all patients want to be responsible for (or involved in) negotiation with their clinician regarding life expectancy disclosure. Given that family-centric medical interactions and decision-making is common in Japan, there is a need to explore how to accommodate overlapping preferences for doctor-, family- and patient-determined prognosis disclosure approaches. Patient education and strategies such as patient question prompt lists may assist patients in understanding that they have right to initiate discussions regarding life expectancy, and equip them with the necessary skills to do this [58]. However, studies have also shown that question prompt lists are most effective when endorsed by the clinician,[59] therefore, it is important to target the communication skills of the both patients and clinicians [60]. Japanese models of communication skills training for oncology specialists (e.g. the ‘SHARE’ staged model for breaking bad news to cancer patients in a preference sensitive way [61]), have had a limited focus on radiation oncologists [62]. There is a need for further research assessing and addressing radiation oncologists’ skills in negotiating patients’ preferences for the timing, content and approach to life expectancy disclosure [60, 62].

### Conclusion

High levels of agreement between patients and clinicians regarding type and time since diagnosis supports the reliability of patient-reported disease information in survey research. Poor agreement between patients’ and radiation oncologists’ perceptions of life expectancy discussions suggests a need for improved patient-clinician communication. There is a need to explore how to accommodate overlapping patient preferences for doctor-, family- and patient-determined prognosis disclosure in Japan.

## Supporting information

S1 File Patient survey (forward and backward IQOLA translation). (DOCX)

S2 File Clinician survey (English). (DOC)

S3 File Clinician survey (Japanese). (DOCX)

S4 File STROBE statement—Checklist of items that should be included in reports of cross-sectional studies. (DOC)

S1 Table Classification of patients’ and clinicians’ non-mutually exclusive preferences for life expectancy disclosure. (DOCX)
