# Supplementary material for: Agreement between patients’ and radiation oncologists’ cancer diagnosis and prognosis perceptions: A cross sectional study in Japan
Source: PLoS One. 2018 Jun 8;13(6):e0198437. doi: 10.1371/journal.pone.0198437 (PMC5993258; doi:10.1371/journal.pone.0198437)
Supplement: S1 File — (DOCX) [file pone.0198437.s001.docx]

**S1 File. Patient survey (forward and backward IQOLA translation).**

Translation Template into JAPANESE of the survey: PATIENT_SURVEY_PILOT

English: **Your views are important to us**

JAPANESE:


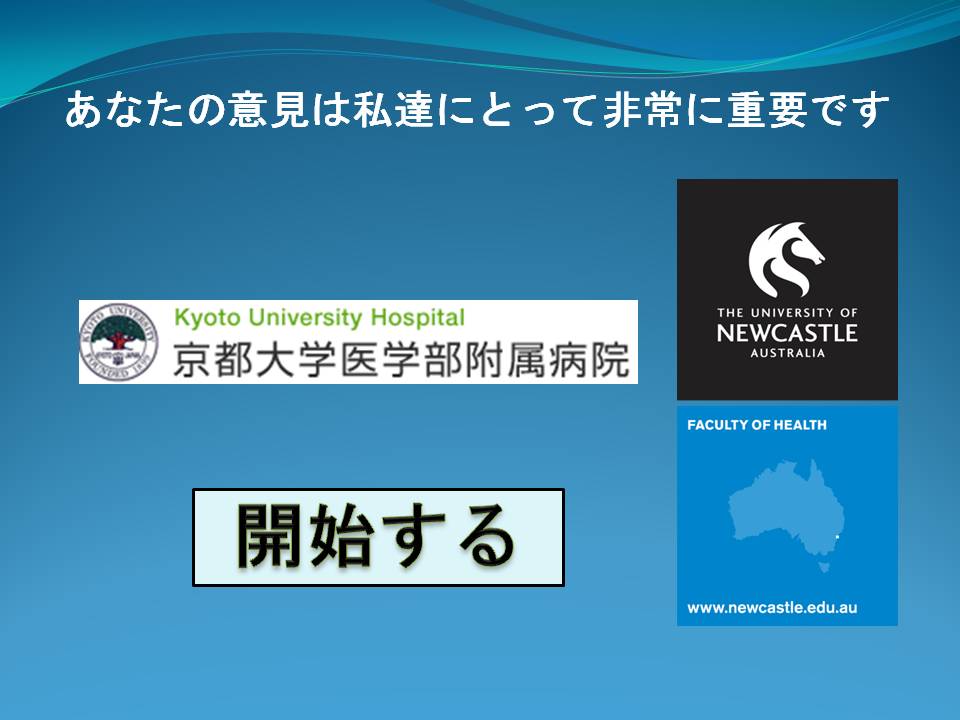


(Do not translate this line: Question Number = 001, Question Name = ID_number)

English: Enter 7 digit ID and then touch “NEXT”

JAPANESE: ７桁のIDを入力し、「次へ」にタッチしてください

English: Please insert participant identification number:

JAPANESE: 参加者のIDを記入してください:

(Do not translate this line: Question Number = 002, Question Name = Introduction)

English: Please touch "NEXT" to begin

JAPANESE: 「次へ」をタッチし、開始してください

English: Your views are important to us


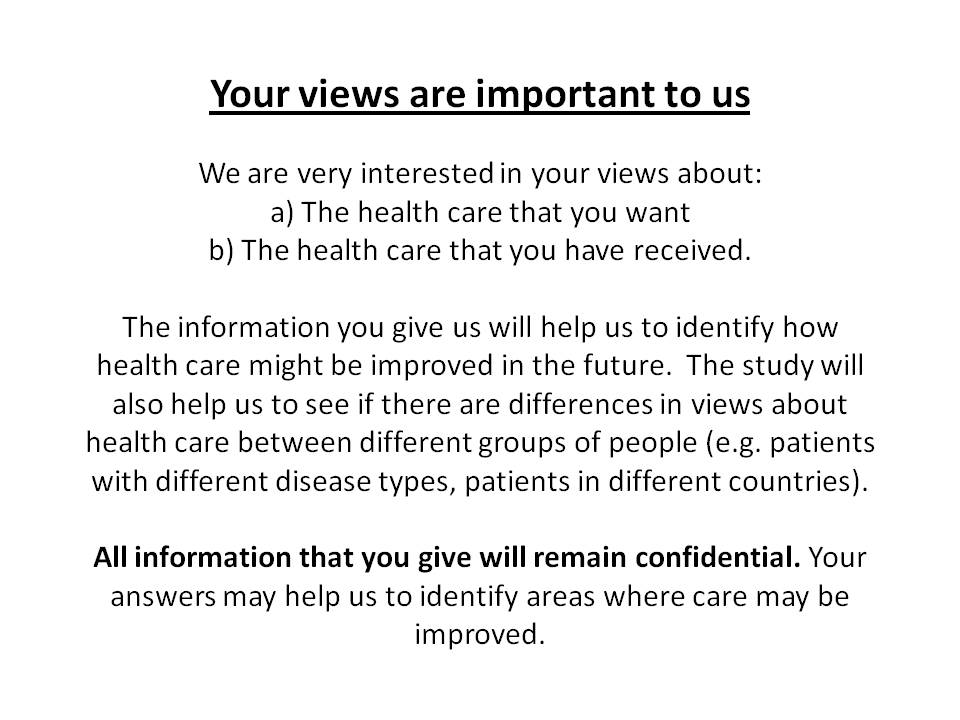


JAPANESE: あなたの意見は私達にとって非常に重要です


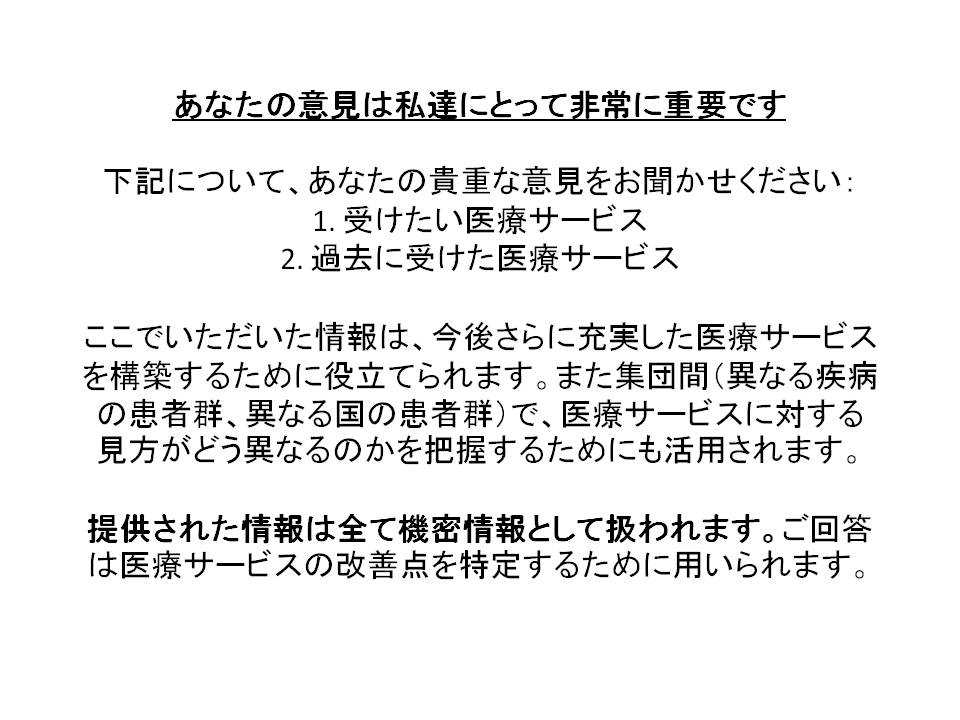


(Do not translate this line: Question Number = 003, Question Name = About_you)

English: Please touch "NEXT" to continue

JAPANESE: 「次へ」をタッチし、続けてください

English: About you


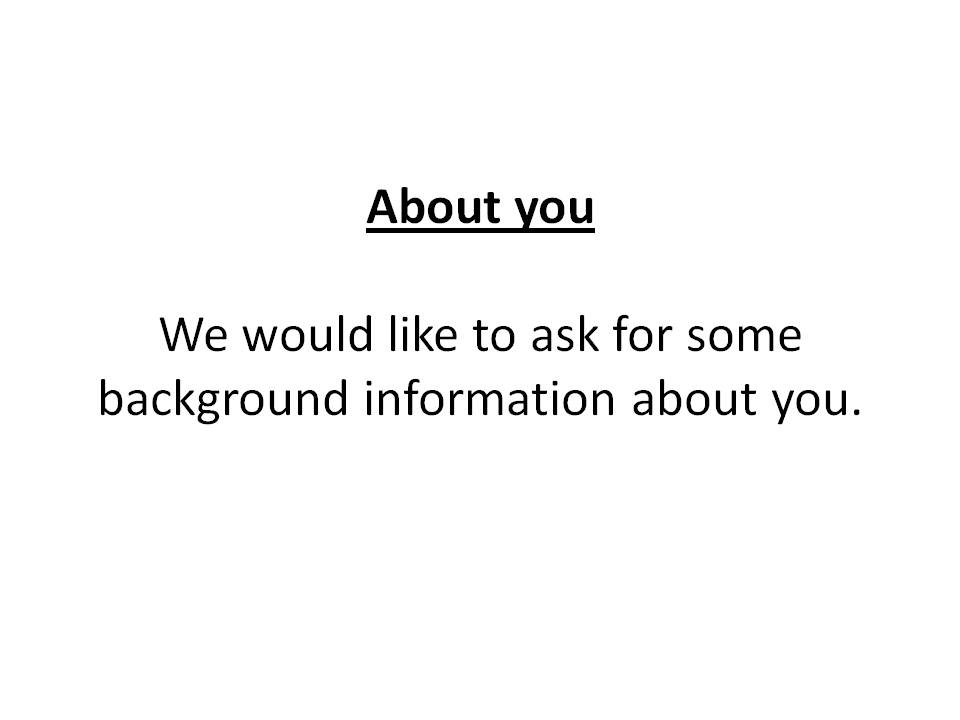


JAPANESE: あなたについて


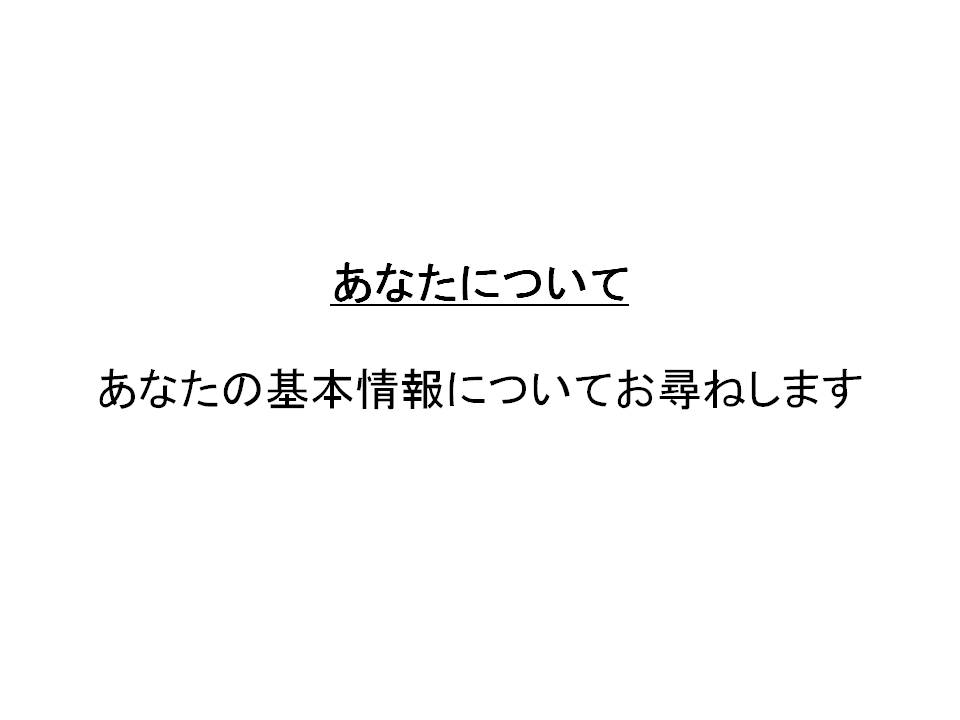


(Do not translate this line: Question Number = 004, Question Name = Gender)

English: Please touch your response:

JAPANESE: 回答にタッチしてください：

English: Are you male or female?

JAPANESE: あなたは男性ですか、女性ですか？

English: Male

JAPANESE: 男性

English: Female

JAPANESE: 女性

(Do not translate this line: Question Number = 005, Question Name = Age)

English: Please type in your age and then touch "NEXT"

JAPANESE: あなたの年齢を入力し、「次へ」をタッチしてください

English: How old are you?

JAPANESE: あなたの年齢は？

(Do not translate this line: Question Number = 006, Question Name = Country)

English: Please touch your response:

JAPANESE: 回答にタッチしてください：

English: What country were you born in?

JAPANESE: あなたの出身国は？

English: Japan

JAPANESE: 日本

English: Other (Please specify)

JAPANESE: その他（国名を入力）

(Do not translate this line: Question Number = 007, Question Name = Other_country)

English: Please type in your response and then touch "NEXT"

JAPANESE: 回答を入力し、「次へ」をタッチしてください

English: What country were you born in?

JAPANESE: あなたの出身国は？

(Do not translate this line: Question Number = 008, Question Name = Education)

English: Please type in your response and then touch "NEXT"

JAPANESE: 回答を入力し、「次へ」をタッチしてください

English: How many years of formal education have you completed?

JAPANESE: 学校での修学年数は？


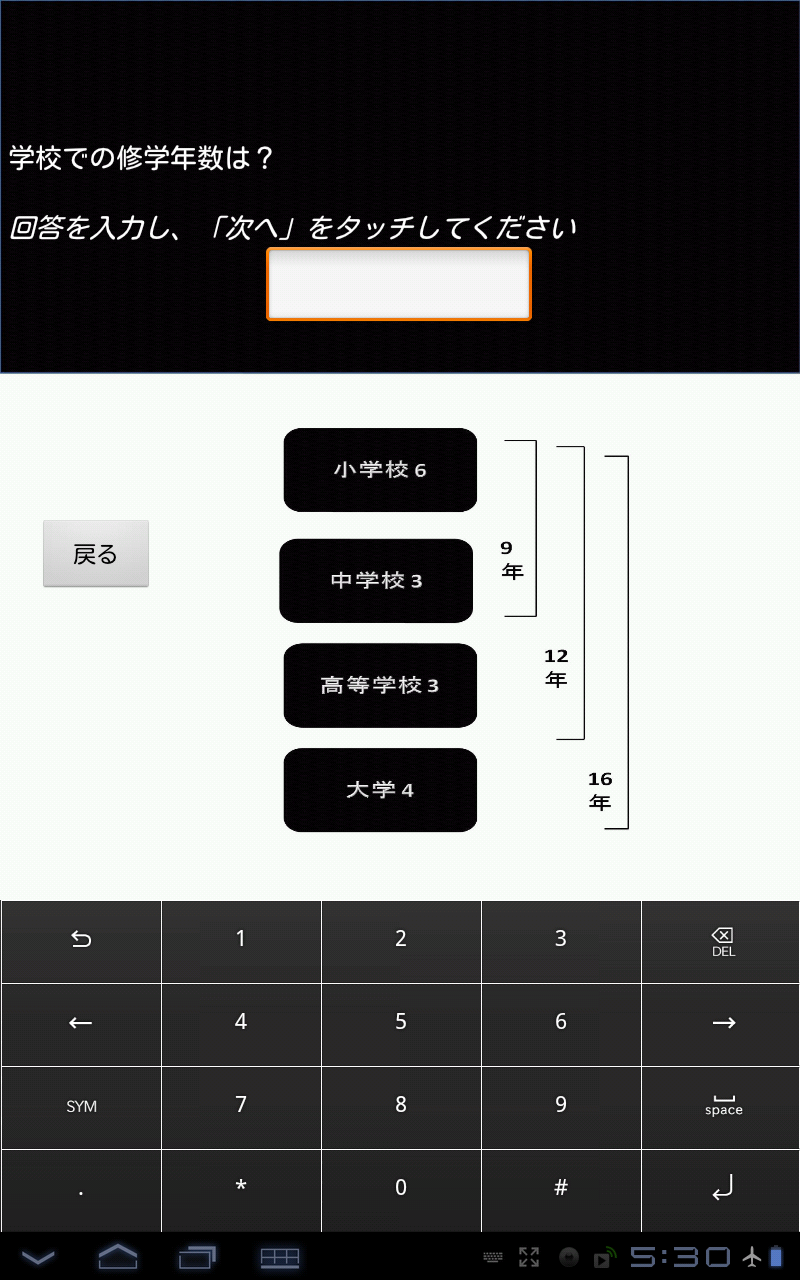


(Do not translate this line: Question Number = 009, Question Name = Employment)

English: Please select the option that best describes your employment situation, and then touch “NEXT”

JAPANESE: 現在の就業状態に最も近い選択肢を選んで、「次へ」をタッチしてください。

English: What is your current employment situation?

JAPANESE: 現在の就業状態は？

English: Full time work

JAPANESE: 正社員

English: Part time work

JAPANESE: パートタイム

English: Paid/ unpaid sick leave

JAPANESE: 有給／無給病気休暇中

English: Permanently unable to work due to illness

JAPANESE: 病気によりに就労不可能 ( 復帰の見込みなし )

English: Home duties

JAPANESE: 家事

English: Retired

JAPANESE: 退職者

English: Unemployed

JAPANESE: 失業中

English: Student

JAPANESE: 学生

(Do not translate this line: Question Number = 010, Question Name = Living_arrangement)

English: Please select all that apply, and then touch “NEXT”

JAPANESE: 該当するものをすべて選び、「次へ」をタッチしてください

English: Who lives with you?

JAPANESE: あなたと同居しているのはどなたですか？

English: My husband/wife/partner

JAPANESE: 夫／妻／パートナー

English: My child/children and/or stepchild/children

JAPANESE: 子供／継子（けいし）

English: Other family

JAPANESE: 他の家族

English: A friend or friends

JAPANESE: 友人

English: An unrelated flat mate or co-tenant

JAPANESE: 家族以外のルームメイトまたは同居人

English: I live alone

JAPANESE: 独居

(Do not translate this line: Question Number = 011, Question Name = Inpatient_status)

English: Please select all that apply, and then touch “NEXT”

JAPANESE: 該当するものをすべて選び、「次へ」をタッチしてください

English: Have you stayed any nights away from your usual place of residence in order to receive radiation therapy treatment?

JAPANESE: 放射線治療のため、自宅以外に宿泊したことはありますか（現在自宅以外に宿泊していますか）？

English: Yes, I have been an inpatient

JAPANESE: はい、入院したことがあります（現在入院中です）

English: Yes, I have stayed in paid accommodation close to the hospital

JAPANESE: はい、病院の近くの有償の宿泊施設に宿泊したことがあります

English: Yes, I have stayed with family/friends close to the hospital

JAPANESE: はい、病院の近くの親族や友人の家に宿泊したことがあります

English: No, I have always stayed at home

JAPANESE: いいえ、すべて自宅から通いました

(Do not translate this line: Question Number = 012, Question Name = Regular_company)

English: Please touch your response:

JAPANESE: 回答にタッチしてください：

English: Do you have a family member or friend who regularly attend your appointments at this clinic with you?

JAPANESE: 放射線治療にいつも付き添ってくれる家族、友人はいますか？

English: Yes

JAPANESE: はい

English: No

JAPANESE: いいえ

(Do not translate this line: Question Number = 013, Question Name = Regular_company2)

English: Please select all that apply, and then touch “NEXT”

JAPANESE: 該当するものをすべて選び、「次へ」をタッチしてください

English: Who regularly attends your appointments at this clinic with you?

JAPANESE: いつも放射線治療に付き添ってくれるのは誰ですか？

English: Husband/Wife/Partner

JAPANESE: 夫／妻／パートナー

English: Child

JAPANESE: 子供

English: Sibling

JAPANESE: 兄弟／姉妹

English: Parent

JAPANESE: 親

English: Friend

JAPANESE: 友人

English: Other

JAPANESE: その他

(Do not translate this line: Question Number = 014, Question Name = Company_today)

English: Please select all that apply, and then touch “NEXT”

JAPANESE: 該当するものをすべて選び、「次へ」をタッチしてください

English: Do you have a family member or friend with you at your appointment today?

JAPANESE: 今日の放射線治療は家族や友人が付き添ってくれましたか？

English: Yes, Husband/Wife/Partner

JAPANESE: はい、夫／妻／パートナー

English: Yes, Child

JAPANESE: はい、子供

English: Yes, Sibling

JAPANESE: はい、兄弟／姉妹

English: Yes, Parent

JAPANESE: はい、親

English: Yes, Friend

JAPANESE: はい、友人

English: Yes, Other

JAPANESE: はい、その他

English: No

JAPANESE: いいえ

(Do not translate this line: Question Number = 015, Question Name = About_disease)

English: Please touch "NEXT" to continue

JAPANESE: 「次へ」をタッチし、続けてください

English: About your disease


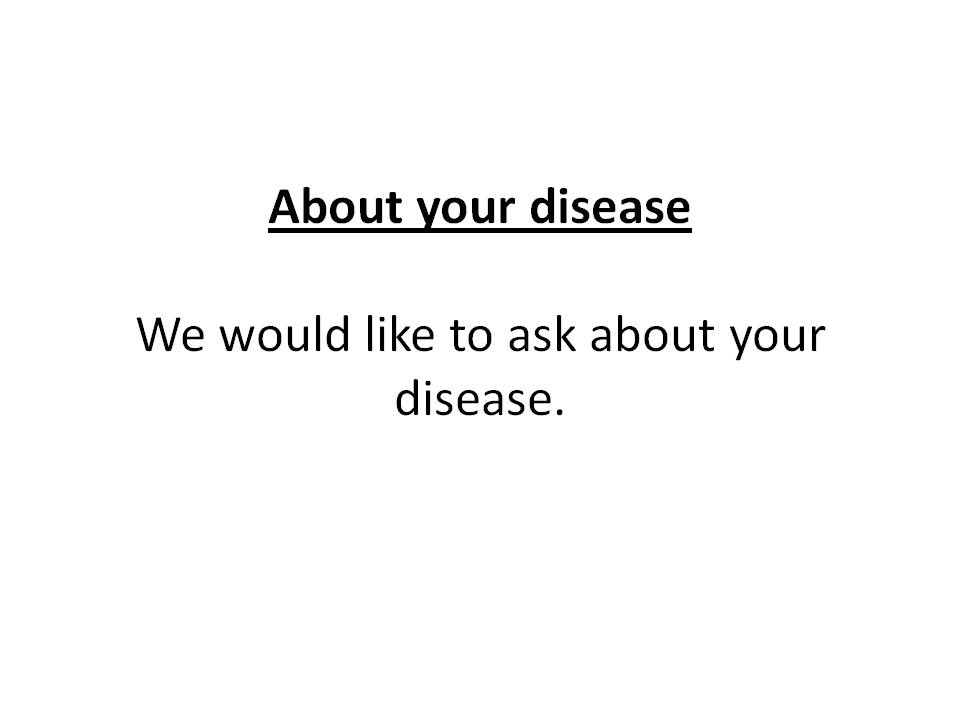


JAPANESE: あなたの病気


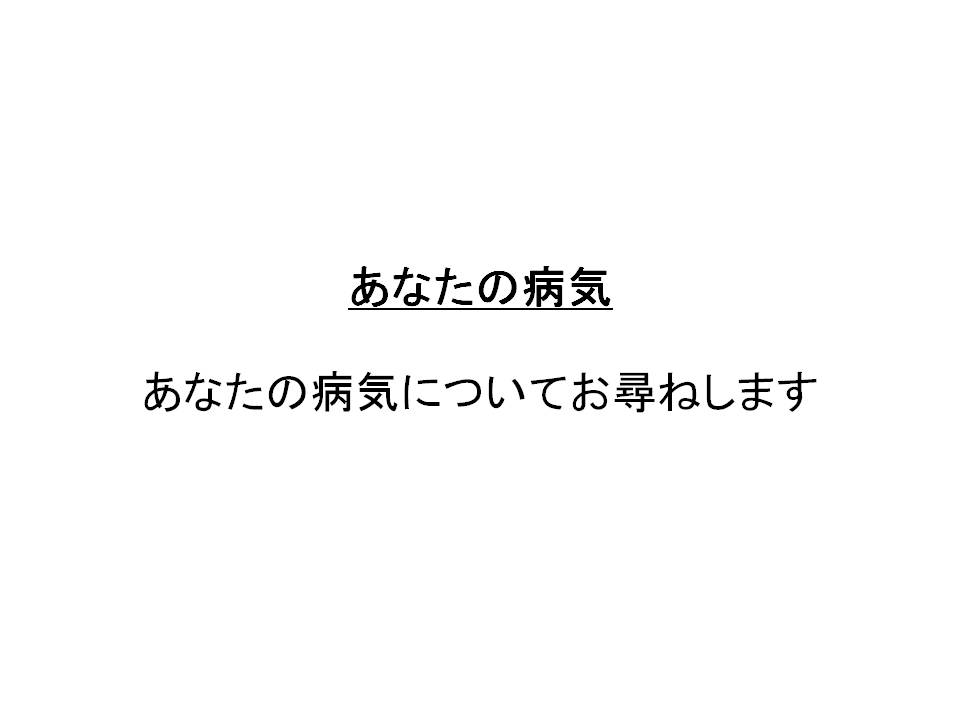


(Do not translate this line: Question Number = 016, Question Name = No_treatments)

English: Please type in your response and then touch "NEXT"

JAPANESE: 回答を入力し、「次へ」をタッチしてください

English: How many radiotherapy treatments have you had (not including today)?

JAPANESE: これまでに何回放射線治療を受けましたか（本日の治療は含めません）

(Do not translate this line: Question Number = 017, Question Name = Know_diagnosis)

English: Please touch your response:

JAPANESE: 回答にタッチしてください：

English: Do you know your diagnosis?

JAPANESE: ご自分の診断名をご存知ですか

English: Yes

JAPANESE: はい

English: No

JAPANESE: いいえ

(Do not translate this line: Question Number = 018, Question Name = Diagnosis_discovery)

English: Please touch your response:

JAPANESE: 回答にタッチしてください：

English: How did you find out about your diagnosis?

JAPANESE: 診断名をどのようにして知りましたか？

English: From my doctor

JAPANESE: 医師から

English: From my family

JAPANESE: 家族から

English: Other

JAPANESE: その他

(Do not translate this line: Question Number = 019, Question Name = Cancer_type)

English: What type of cancer do you have? If you have had more than one type of cancer, please touch your most recent primary cancer. Please tick only one box below.

JAPANESE: あなたのがんの種類は？ 2種以上のがんが併発している場合は、一番最近に 診断された原発性のがんを下記から選んでチェックしてください。

English: Breast

JAPANESE: 乳がん

English: Colorectal

JAPANESE: 結腸直腸がん

English: Prostate

JAPANESE: 前立腺がん

English: Lung

JAPANESE: 肺がん

English: Melanoma

JAPANESE: 悪性黒色腫

English: Don’t know

JAPANESE: わかりません

English: Other, please specify

JAPANESE: その他（具体的に入力してください）

(Do not translate this line: Question Number = 020, Question Name = Other_cancer)

English: Please type in your response and then touch "NEXT"

JAPANESE: 回答を入力し、「次へ」をタッチしてください

English: What type of cancer do you have?

JAPANESE: あなたのがんの種類は？

(Do not translate this line: Question Number = 021, Question Name = Year_diagnosed)

English: Please type in your response and then touch "NEXT"

JAPANESE: 回答を入力し、「次へ」をタッチしてください

English: In what year were you diagnosed with the cancer for which you are currently receiving care (Christian year)? If you have had more than one diagnosis, please think about your most recent primary cancer when answering.

JAPANESE: がんの診断を受けたのはいつですか？西暦でお答えください。がんの診断が2回以上にわたる場合は、 一番最近の原発性のがんを想定して答えてください。

(Do not translate this line: Question Number = 022, Question Name = Month_diagnosed)

English: Please touch your response:

JAPANESE: 回答にタッチしてください：

English: In what month of <Year_diagnosed> were you diagnosed with the cancer for which you are currently receiving care?

JAPANESE: がんの診断を受けたのはいつですか？　<Year_diagnosed>年の何月かをお答えください。

English: January

JAPANESE: １月

English: February

JAPANESE: ２月

English: March

JAPANESE: ３月

English: April

JAPANESE: ４月

English: May

JAPANESE: ５月

English: June

JAPANESE: ６月

English: July

JAPANESE: ７月

English: August

JAPANESE: ８月

English: September

JAPANESE: ９月

English: October

JAPANESE: １０月

English: November

JAPANESE: １１月

English: December

JAPANESE: １２月

(Do not translate this line: Question Number = 023, Question Name = Treatment_aim)

English: Please touch your response:

JAPANESE: 回答にタッチしてください：

English: What do you understand to be the main aim of your current treatment?

JAPANESE: 今の治療の主な目的についてどのように理解していますか？

English: To cure the cancer

JAPANESE: がんの治癒

English: To prevent the cancer from coming back

JAPANESE: がん再発の防止

English: To control symptoms of cancer (cure is not possible)

JAPANESE: がんに伴う症状のコントロール（治癒は不可能）

(Do not translate this line: Question Number = 024, Question Name = Surgery)

English: Please touch your response:

JAPANESE: 回答にタッチしてください：

English: Have you had surgery to treat the cancer?

JAPANESE: がんの治療にあたって手術を受けましたか？

English: Yes

JAPANESE: はい

English: No

JAPANESE: いいえ

English: No, but planning to have surgery

JAPANESE: いいえ、ただしこれから受ける予定です

English: Don’t know

JAPANESE: わかりません

(Do not translate this line: Question Number = 025, Question Name = Chemo)

English: Please touch your response:

JAPANESE: 回答にタッチしてください：

English: Have you had chemotherapy to treat the cancer?

JAPANESE: がんの治療にあたって化学療法は受けましたか？

English: Yes

JAPANESE: はい

English: No

JAPANESE: いいえ

English: No, but planning to have chemotherapy

JAPANESE: いいえ、ただしこれから受ける予定です

English: Don’t know

JAPANESE: わかりません

(Do not translate this line: Question Number = 026, Question Name = About_life_expectancy)

English: Please touch your response:

JAPANESE: 回答にタッチしてください：

English: Discussions about Life Expectancy

The following questions ask for your views about how your doctor should approach discussions

about whether cancer will affect the length of your life (your life expectancy).

This will provide the research team and clinicians with information that may enable us to

further improve services for cancer patients. If you are not willing to complete this section,

please touch “Please skip to the next section of the survey”.

If you choose to answer these questions, you will still have the option of skipping

the rest of the life expectancy section available to you on each question screen.

JAPANESE: 余命に関する話し合い


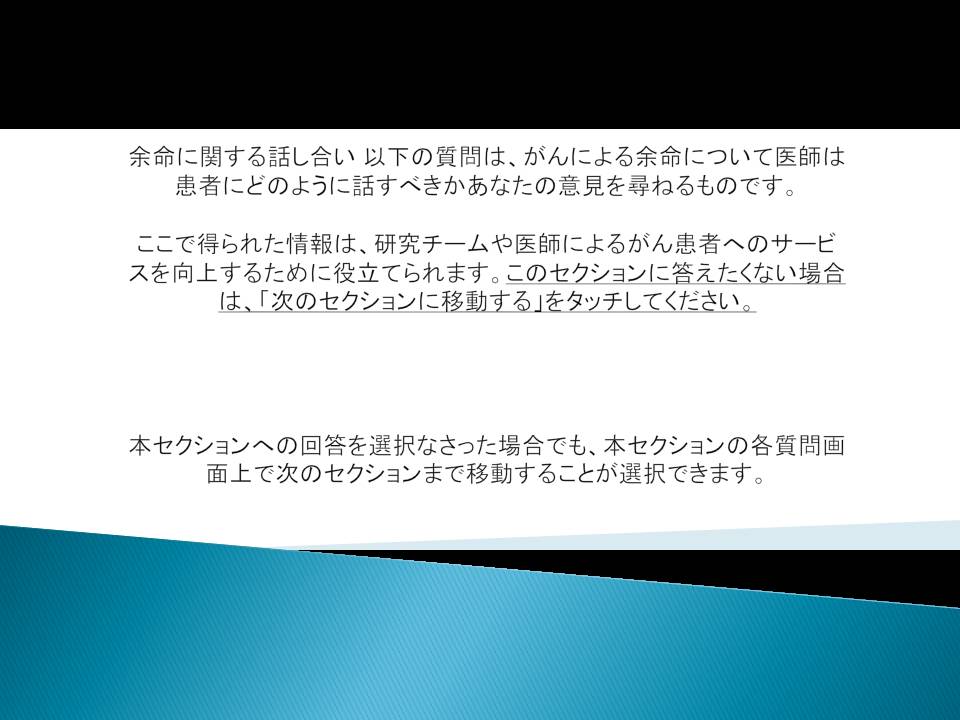


English: I am willing to complete this section of the survey

JAPANESE: このセクションに回答します

English: Please skip to the next section of the survey

JAPANESE: 次のセクションまでスキップします

(Do not translate this line: Question Number = 027, Question Name = Prefs_LE)

English: Please indicate how much you agree with the following statements and then touch “NEXT”

JAPANESE: 下記の文にどの程度同意できるかを選択し、「次へ」をタッチしてください。

English: When discussing life expectancy, I would prefer my radiation oncologist to:

JAPANESE: 放射線腫瘍医と余命について話し合うとき、次のことを希望します

English: Ask me if I want to discuss life expectancy

JAPANESE: 私自身が余命について話し合いを希望するかどうか尋ねてほしい

English: Just tell me the news he/she thinks I can cope with

JAPANESE: 医師には私が対処できると思われる情報だけを伝えてほしい

English: Just tell me the good news

JAPANESE: 良い内容だけにしてほしい

English: Tell me everything he/she can

JAPANESE: 医師が知っていることをすべて話してほしい

English: Tell my partner or family the news and let them decide whether I should be told

JAPANESE: 家族やパートナーに情報を伝えて、彼らに私に伝えるかどうかは判断させてほしい

English: Strongly disagree

JAPANESE: 全く希望しない

English: Disagree

JAPANESE: 希望しない

English: Agree

JAPANESE: 希望する

English: Strongly agree

JAPANESE: 強く希望する

(Do not translate this line: Question Number = 028, Question Name = Talked_about_le)

English: Please touch your response:

JAPANESE: 回答にタッチしてください：

English: Have you and your radiation oncologist talked about your life expectancy?

JAPANESE: あなたとあなたの主治医である放射線腫瘍医と 余命について話したことはありますか

English: Yes

JAPANESE: はい

English: No

JAPANESE: いいえ

(Do not translate this line: Question Number = 029, Question Name = Initiation_le)

English: Please touch your response:

JAPANESE: 回答にタッチしてください：

English: How did the discussion about life expectancy begin?

JAPANESE: 余命に関する話は何をきっかけに始まりましたか？

English: I asked my doctor if we could talk about it

JAPANESE: わたしが余命についての話を希望しました

English: My doctor asked me if I wanted to talk about it

JAPANESE: 医師は、私が余命について話し合いたいかどうか尋ねました

English: My doctor discussed it without asking me first

JAPANESE: 医師は、私が余命について話し合いたいかどうか確認せずに話し始めました

English: Other

JAPANESE: その他

(Do not translate this line: Question Number = 030, Question Name = Current_LE)

English: Please touch your response:

JAPANESE: 回答にタッチしてください：

English: While my radiation oncologist cannot be certain, he/she has suggested that currently:

JAPANESE: 担当の放射線腫瘍医は、確実ではないとしながらも、 現在の可能性として以下を提示しました：

English: My cancer diagnosis will not affect my life expectancy

JAPANESE: あなたのがんは余命には影響を与えないと思われます

English: It is far too early to tell

JAPANESE: 余命について話し合いをするにはまだ早すぎます

English: I will live more than 5 years

JAPANESE: あなたの余命は5年以上でしょう

English: I will live for 2-5 years

JAPANESE: あなたの余命は２～５年でしょう

English: I will live for less than 2 years

JAPANESE: あなたの余命は2年に満たないでしょう

(Do not translate this line: Question Number = 031, Question Name = Want_to_discuss_LE)

English: Please touch your response:

JAPANESE: 回答にタッチしてください：

English: Would you like to talk to your doctor about your life expectancy?

JAPANESE: あなたは余命について医師に相談したいと思いますか.

English: Yes

JAPANESE: はい

English: No

JAPANESE: いいえ

(Do not translate this line: Question Number = 032, Question Name = HADS Info)

English: Please read the text below and then touch "NEXT" to continue.

JAPANESE: 下記の文章を読み、「次へ」をタッチし、続けてください。

English: HADS


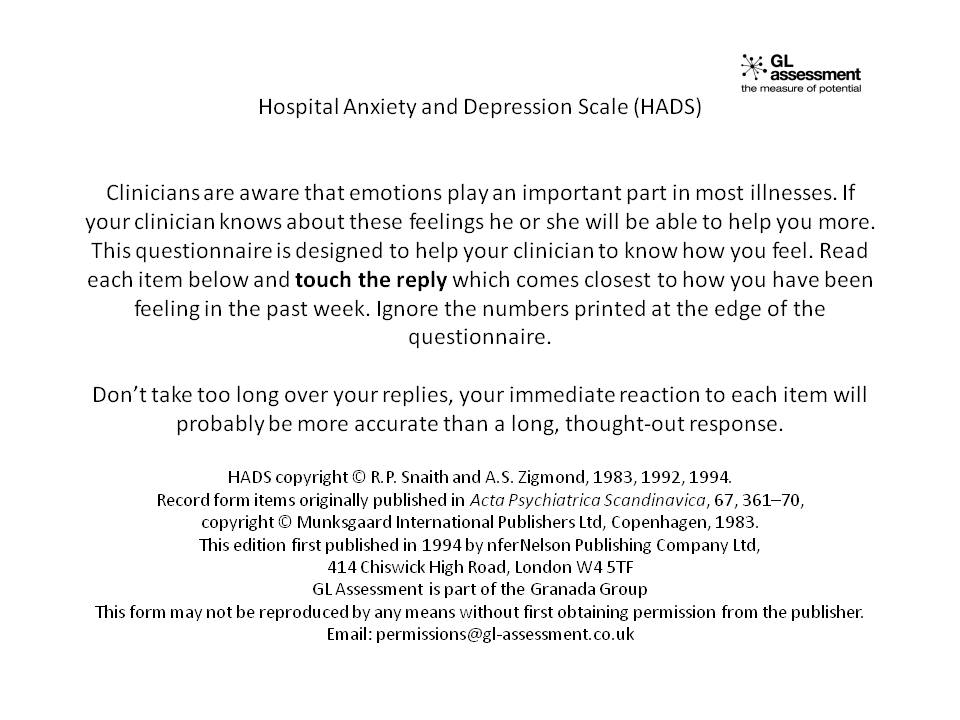


JAPANESE: 病院における不安と
抑うつに関する質問票 (HADS)


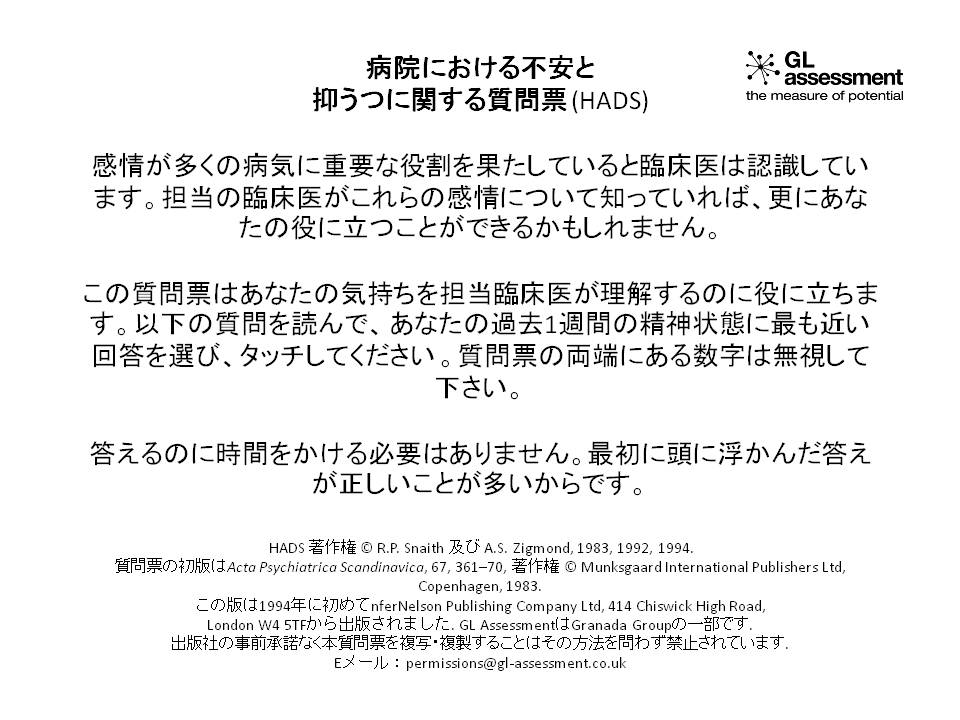


(Do not translate this line: Question Number = 033, Question Name = HADS1)

English: Touch the reply which comes closest to how you have been feeling in the past week

JAPANESE: あなたの過去1週間の精神状態に最も近い回答を選び、タッチしてください

English: I feel tense or ‘wound up’

JAPANESE: 緊張感を感じましたか？

English: Most of the time

JAPANESE: ほとんどいつも感じた

English: A lot of the time

JAPANESE: しばしば感じた

English: From time to time, occasionally

JAPANESE: 時々感じた

English: Not at all

JAPANESE: 全く感じなかった

(Do not translate this line: Question Number = 034, Question Name = HADS2)

English: Touch the reply which comes closest to how you have been feeling in the past week

JAPANESE: あなたの過去1週間の精神状態に最も近い回答を選び、タッチしてください

English: I still enjoy the things I used to enjoy

JAPANESE: 以前楽しんでいたことを変わらず楽しめましたか？

English: Definitely as much

JAPANESE: 以前と全く同じように楽しめた

English: Not quite so much

JAPANESE: 以前ほど楽しめなかった

English: Only a little

JAPANESE: 少ししか楽しめなかった

English: Hardly at all

JAPANESE: 全く楽しめなかった

(Do not translate this line: Question Number = 035, Question Name = HADS3)

English: Touch the reply which comes closest to how you have been feeling in the past week

JAPANESE: あなたの過去1週間の精神状態に最も近い回答を選び、タッチしてください

English: I get a sort of frightened feeling as if something awful is about to happen

JAPANESE: 何かひどいことが今にも起こりそうな 恐怖感を感じましたか？

English: Very definitely and quite badly

JAPANESE: かなり強く感じた

English: Yes, but not too badly

JAPANESE: 感じたがあまり強くはなかった

English: A little, but it doesn’t worry me

JAPANESE: わずかに感じたが気にならなかった

English: Not at all

JAPANESE: 全く感じなかった

(Do not translate this line: Question Number = 036, Question Name = HADS4)

English: Touch the reply which comes closest to how you have been feeling in the past week

JAPANESE: あなたの過去1週間の精神状態に最も近い回答を選び、タッチしてください

English: I can laugh and see the funny side of things

JAPANESE: 物事を面白いと感じることができ、笑うことができましたか？

English: As much as I always could

JAPANESE: 以前と同じように笑えた

English: Not quite so much now

JAPANESE: 以前ほどは笑えなかった

English: Definitely not so much now

JAPANESE: 明らかに以前より笑えなかった

English: Not at all

JAPANESE: 全く笑えなかった

(Do not translate this line: Question Number = 037, Question Name = HADS5)

English: Touch the reply which comes closest to how you have been feeling in the past week

JAPANESE: あなたの過去1週間の精神状態に最も近い回答を選び、タッチしてください

English: Worrying thoughts go through my mind

JAPANESE: 不安が胸をよぎりましたか？

English: A great deal of the time

JAPANESE: ほとんどいつもよぎった

English: A lot of the time

JAPANESE: しばしばよぎった

English: Not too often

JAPANESE: 時々よぎった

English: Very little

JAPANESE: ほとんどよぎらなかった

(Do not translate this line: Question Number = 038, Question Name = HADS6)

English: Touch the reply which comes closest to how you have been feeling in the past week

JAPANESE: あなたの過去1週間の精神状態に最も近い回答を選び、タッチしてください

English: I feel cheerful

JAPANESE: 明るい気分でしたか？

English: Never

JAPANESE: 明るい気分の時は全くなかった

English: Not often

JAPANESE: 明るい気分の時はあまりなかった

English: Sometimes

JAPANESE: 時々明るい気分だった

English: Most of the time

JAPANESE: ほとんどいつも明るい気分だった

(Do not translate this line: Question Number = 039, Question Name = HADS7)

English: Touch the reply which comes closest to how you have been feeling in the past week

JAPANESE: あなたの過去1週間の精神状態に最も近い回答を選び、タッチしてください

English: I can sit at ease and feel relaxed

JAPANESE: のんびりリラックスして、くつろぐことができましたか？

English: Definitely

JAPANESE: 常にできた

English: Usually

JAPANESE: たいていできた

English: Not often

JAPANESE: あまりできなかった

English: Not at all

JAPANESE: 全くできなかった

(Do not translate this line: Question Number = 040, Question Name = HADS8)

English: Touch the reply which comes closest to how you have been feeling in the past week

JAPANESE: あなたの過去1週間の精神状態に最も近い回答を選び、タッチしてください

English: I feel as if I am slowed down

JAPANESE: 頭の回転や動作が鈍くなったように感じましたか？

English: Nearly all the time

JAPANESE: ほとんどいつもそう感じた

English: Very often

JAPANESE: しばしばそう感じた

English: Sometimes

JAPANESE: 時々そう感じた

English: Not at all

JAPANESE: 全くそう感じなかった

(Do not translate this line: Question Number = 041, Question Name = HADS9)

English: Touch the reply which comes closest to how you have been feeling in the past week

JAPANESE: あなたの過去1週間の精神状態に最も近い回答を選び、タッチしてください

English: I get a sort of frightened feeling like ‘butterflies’ in the stomach

JAPANESE: 胃が締め付けられるような恐怖感がありましたか？

English: Not at all

JAPANESE: 全くなかった

English: Occasionally

JAPANESE: 時々あった

English: Quite often

JAPANESE: しばしばあった

English: Very often

JAPANESE: ほとんどいつもあった

(Do not translate this line: Question Number = 042, Question Name = HADS10)

English: Touch the reply which comes closest to how you have been feeling in the past week

JAPANESE: あなたの過去1週間の精神状態に最も近い回答を選び、タッチしてください

English: I have lost interest in my appearance

JAPANESE: 自分の身なりにかまわなくなっていましたか？

English: Definitely

JAPANESE: 全くかまわなくなっていた

English: I don’t take as much care as I should

JAPANESE: おろそかにしていた

English: I may not take quite as much care

JAPANESE: いくらかおろそかにしていたかもしれない

English: I take just as much care as ever

JAPANESE: 今までどおり気を配っていた

(Do not translate this line: Question Number = 043, Question Name = HADS11)

English: Touch the reply which comes closest to how you have been feeling in the past week

JAPANESE: あなたの過去1週間の精神状態に最も近い回答を選び、タッチしてください

English: I feel restless as if I have to be on the move

JAPANESE: 落ち着かず、じっとしていられないような感じがしましたか？

English: Very much indeed

JAPANESE: 非常に強くそう感じた

English: Quite a lot

JAPANESE: かなりそう感じた

English: Not very much

JAPANESE: あまりそう感じなかった

English: Not at all

JAPANESE: 全くそう感じなかった

(Do not translate this line: Question Number = 044, Question Name = HADS12)

English: Touch the reply which comes closest to how you have been feeling in the past week

JAPANESE: あなたの過去1週間の精神状態に最も近い回答を選び、タッチしてください

English: I look forward with enjoyment to things

JAPANESE: 物事を楽しみに待つことができましたか？

English: As much as I ever did

JAPANESE: 以前と同じようにできた

English: Rather less than I used to

JAPANESE: 以前ほどはできなかった

English: Definitely less than I used to

JAPANESE: 明らかに以前よりできなかった

English: Hardly at all

JAPANESE: 全くできなかった

(Do not translate this line: Question Number = 045, Question Name = HADS13)

English: Touch the reply which comes closest to how you have been feeling in the past week

JAPANESE: あなたの過去1週間の精神状態に最も近い回答を選び、タッチしてください

English: I get sudden feelings of panic

JAPANESE: 急に恐怖感に襲われることがありましたか？

English: Very often indeed

JAPANESE: ほとんどいつもあった

English: Quite often

JAPANESE: しばしばあった

English: Not very often

JAPANESE: 時々あった

English: Not at all

JAPANESE: 全くなかった

(Do not translate this line: Question Number = 046, Question Name = HADS14)

English: Touch the reply which comes closest to how you have been feeling in the past week

JAPANESE: あなたの過去1週間の精神状態に最も近い回答を選び、タッチしてください

English: I can enjoy a good book or radio or television programme

JAPANESE: 本やラジオ、テレビの番組を楽しめましたか？

English: Often

JAPANESE: たいてい楽しめた

English: Sometimes

JAPANESE: 時々楽しめた

English: Not often

JAPANESE: 楽しめる時はあまりなかった

English: Very seldom

JAPANESE: めったに楽しめなかった

(Do not translate this line: Question Number = 047, Question Name = About_wellbeing)

English: Please touch "NEXT" to continue

JAPANESE: 「次へ」をタッチし、続けてください

English: Perceptions of wellbeing


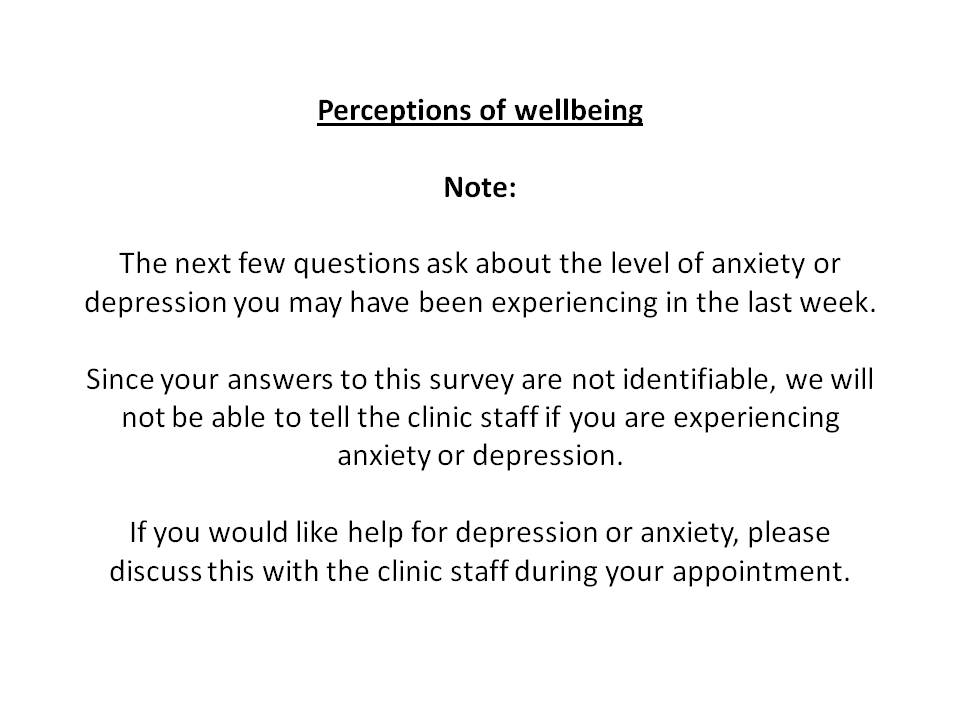


JAPANESE: 精神状態の認識について


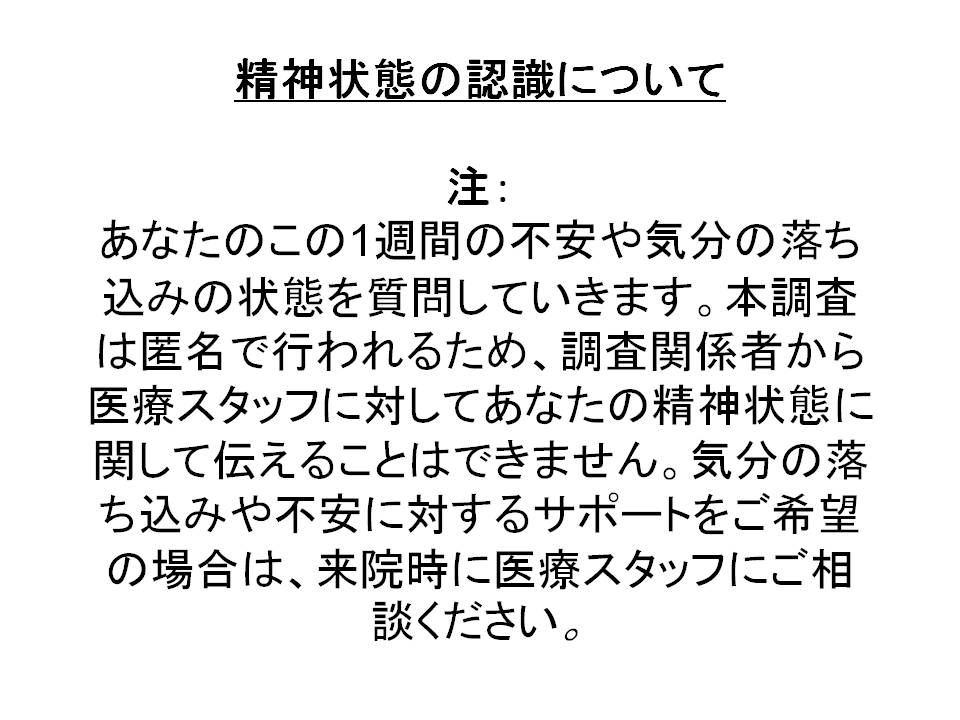


(Do not translate this line: Question Number = 048, Question Name = Perceived_anxiety)

English: Touch the reply which comes closest to how you have been feeling in the past week

JAPANESE: あなたの過去1週間の精神状態に最も近い回答を選び、タッチしてください

English: What level of ANXIETY have you been experiencing IN THE LAST WEEK?

JAPANESE: 過去1週間に感じた不安はどの程度でしたか？

English: No anxiety

JAPANESE: まったくなし

English: Mild anxiety

JAPANESE: 少しの不安（あまり気にならない）

English: Moderate anxiety

JAPANESE: 気になるレベルの不安

English: Severe anxiety

JAPANESE: ひどく不安を感じる

(Do not translate this line: Question Number = 049, Question Name = Perceived_depression)

English: Touch the reply which comes closest to how you have been feeling in the past week

JAPANESE: あなたの過去1週間の精神状態に最も近い回答を選び、タッチしてください

English: What level of DEPRESSION have you been experiencing IN THE LAST WEEK?

JAPANESE: 過去1週間に感じたうつ状態はどの程度でしたか？

English: No depression

JAPANESE: まったく落ち込むことは無かった

English: Mild depression

JAPANESE: 少し落ち込むことはあった（あまり気にならない）

English: Moderate depression

JAPANESE: 気になるレベルの落ち込みを感じた

English: Severe depression

JAPANESE: ひどく落ち込みを感じた

(Do not translate this line: Question Number = 050, Question Name = Psych_support_prefs)

English: Please touch your response:

JAPANESE: 回答にタッチしてください：

English: Given your current levels of ANXIETY AND/OR DEPRESSION, would you like to be offered some professional help?

JAPANESE: 現在の不安やうつ状態を受けて、 専門的なサポートを受けたいと思いますか

English: Yes

JAPANESE: はい

English: No

JAPANESE: いいえ

(Do not translate this line: Question Number = 051, Question Name = Why_no_support)

English: Please touch your response:

JAPANESE: 回答にタッチしてください：

English: Why don’t you want professional support for ANXIETY AND/OR DEPRESSION?

JAPANESE: なぜ不安やうつ状態について専門的なサポートを受けたくないのですか？

English: Not experiencing much anxiety/depression

JAPANESE: あまり不安や落ち込みを感じないから

English: Anxiety/depression is normal for someone in my situation

JAPANESE: わたしのような状況だと、不安に感じたり落ち込んだりするのが普通だと思うから

English: My anxiety/depression is not much higher than usual

JAPANESE: 不安や落ち込みのレベルは普段とくらべてそんなにひどくないから

English: I don’t think professional assistance would help

JAPANESE: 専門的なサポートでは解決できないと思うから

English: My anxiety/depression will reduce once this phase of treatment is over

JAPANESE: 今の治療が終われば不安やうつ状態は軽減すると思うから

(Do not translate this line: Question Number = 052, Question Name = Depression_history)

English: Please touch your response:

JAPANESE: 回答にタッチしてください：

English: Do you have a history of depression?

JAPANESE: 過去にうつ状態になったことはありますか？

English: Yes

JAPANESE: はい

English: No

JAPANESE: いいえ

(Do not translate this line: Question Number = 053, Question Name = Anxiety_history)

English: Please touch your response:

JAPANESE: 回答にタッチしてください：

English: Do you have a history of anxiety?

JAPANESE: 過去に不安を経験したことはありますか？

English: Yes

JAPANESE: はい

English: No

JAPANESE: いいえ

(Do not translate this line: Question Number = 054, Question Name = Acceptability)

English: Please touch "NEXT" to continue

JAPANESE: 「次へ」をタッチし、続けてください

English: Acceptability Module


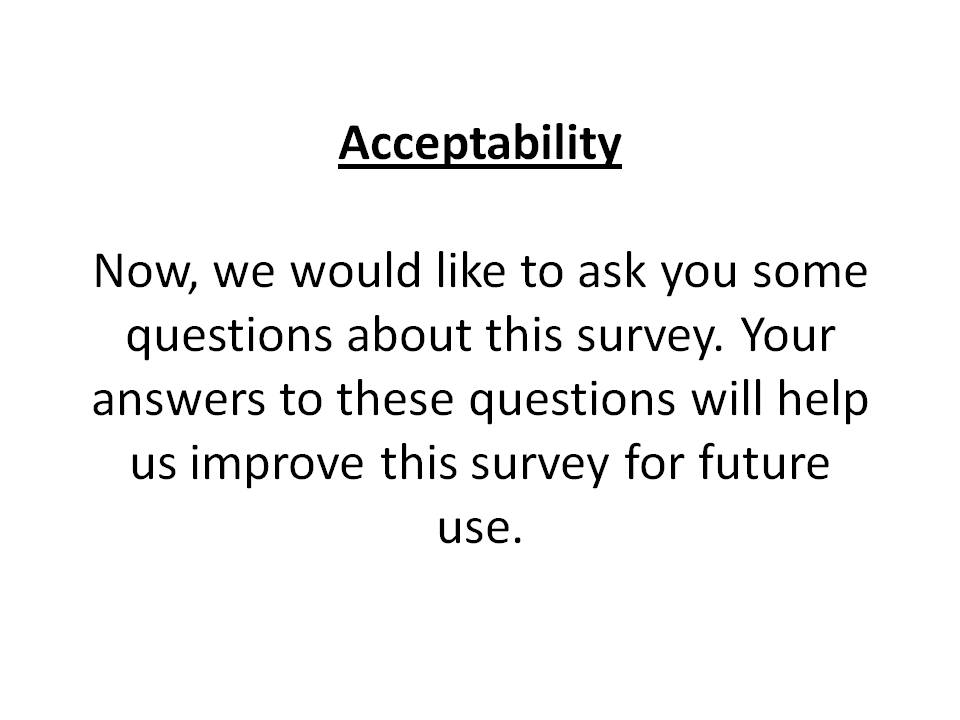


JAPANESE: **本調査の受け入れ度について**

このセクションは本調査に関するあなたの受け入れの程度についての質問です。

ここで提供された回答は、今後さらに本調

査を改善するために活用されます。

(Do not translate this line: Question Number = 055, Question Name = Acceptability1)

English: Please indicate how much you agree with the following statements and then touch “NEXT”

JAPANESE: 下記の文にどの程度同意できるかを選択し、「次へ」をタッチしてください。

English: When completing the survey today:

JAPANESE: 本調査への回答終了後：

English: The instructions were easy to follow

JAPANESE: 質問の指示はわかりやすかった

English: The questions were easy to understand

JAPANESE: 質問は理解しやすかった

English: The touch screen was easy to use

JAPANESE: タッチスクリーンは使いやすかった

English: I had enough time to complete all the questions

JAPANESE: すべての質問に答える時間は十分にあった

English: I felt comfortable answering the questions

JAPANESE: 不快な気持になることなく質問に答えることができた

English: The touchscreen allowed enough privacy

JAPANESE: タッチスクリーンを用いた調査で、プライバシーは十分保護されていた

English: Strongly disagree

JAPANESE: 全くそう思わない

English: Disagree

JAPANESE: そう思わない

English: Agree

JAPANESE: そう思う

English: Strongly agree

JAPANESE: 強くそう思う

(Do not translate this line: Question Number = 056, Question Name = Acceptability2)

English: Please touch your response:

JAPANESE: 回答にタッチしてください：

English: How often would you be willing to complete this survey (with different questions each time) while waiting for radiation therapy?

JAPANESE: 放射線治療の待ち時間中に、どのくらいの頻度で あれば本調査に（毎回質問内容を変え）参加していただけますか？

English: Only once (just this survey)

JAPANESE: 今回の調査のみ

English: Less than half the visits

JAPANESE: 来院回数の半分以下

English: Half of the visits

JAPANESE: 来院回数の半分程度

English: Most visits

JAPANESE: たいていの来院時に可能

English: Every visit

JAPANESE: 来院時はいつでも可能

(Do not translate this line: Question Number = 057, Question Name = Thank you(1))

English:


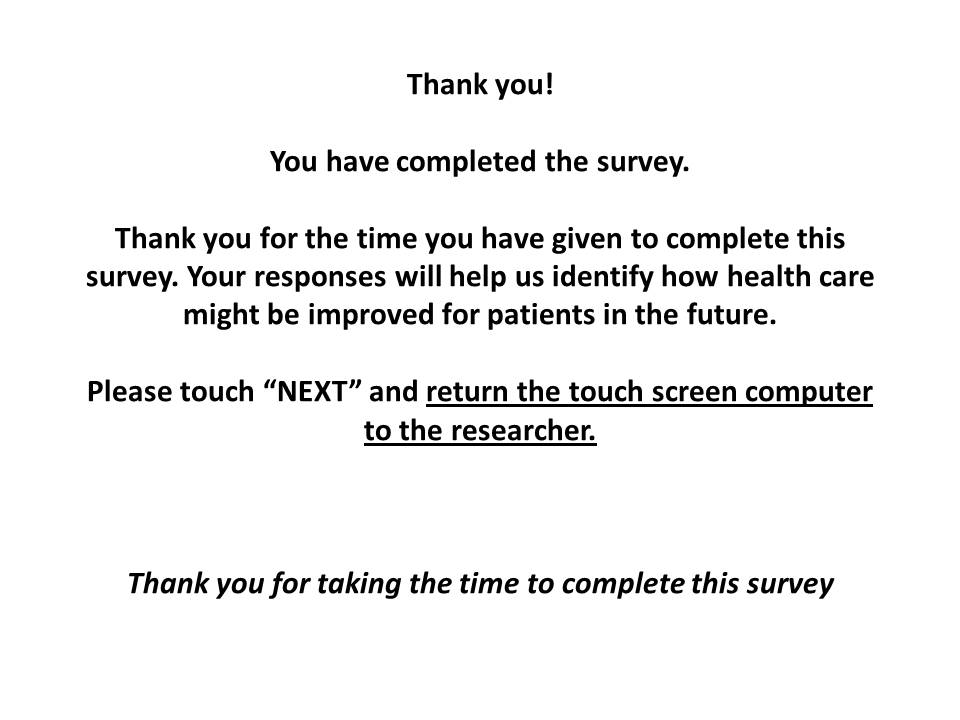


JAPANESE:


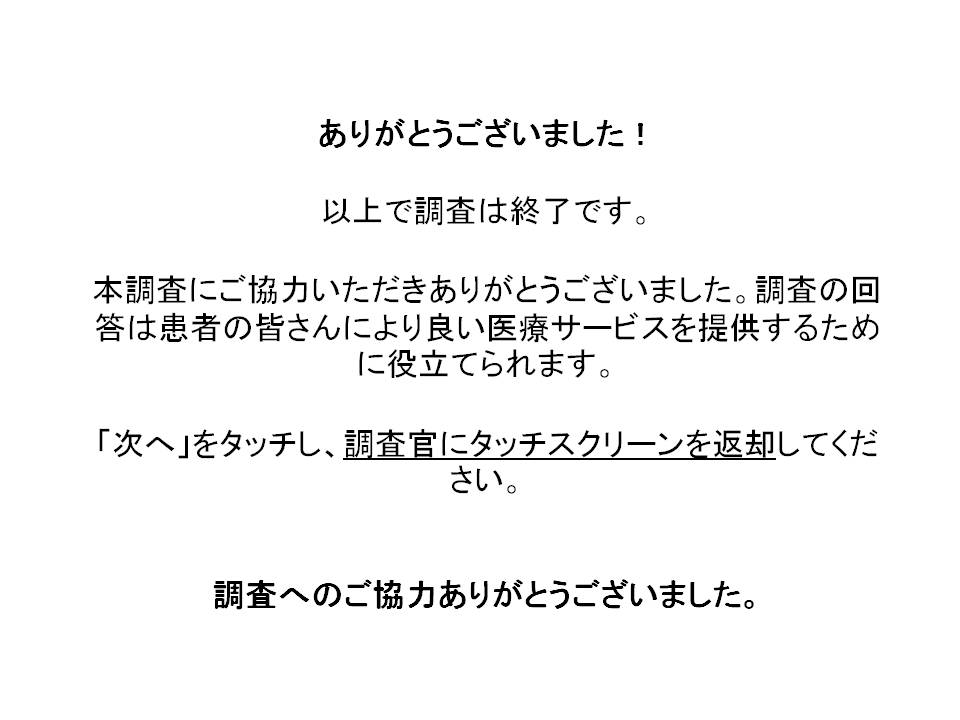


(Do not translate this line: Various)

English: JAPANESE

JAPANESE: 日本語

English: NEXT

JAPANESE: 次へ

English: BACK

JAPANESE: 戻る

English: EXIT SURVEY

JAPANESE: 取り消し

(Do not translate this remark:

Please add your personalized messages on the start screen, end screen, language screen and information screens to this translation template.)
