# Supplementary material for: Agreement between patients’ and radiation oncologists’ cancer diagnosis and prognosis perceptions: A cross sectional study in Japan
Source: PLoS One. 2018 Jun 8;13(6):e0198437. doi: 10.1371/journal.pone.0198437 (PMC5993258; doi:10.1371/journal.pone.0198437)
Supplement: S2 File — (DOC) [file pone.0198437.s002.doc]

**S2 File. Clinician survey (English).**

**What type of cancer does the patient have?**

If they have had more than one type of cancer, please tick their *most recent* primary cancer.

*Please tick only one box below.*

**🗆**Breast

**🗆**Colorectal

**🗆**Prostate

**🗆**Lung

**🗆**Melanoma

**🗆**Don’t know

**🗆**Other, please specify__________________________

**When was the patient diagnosed with cancer?** __________________

If they have had more than one diagnosis, please think about their *most recent* primary cancer when answering.

**What is the main aim of their current treatment?**

- To cure the cancer
- To prevent the cancer from coming back
- To control symptoms of cancer (cure is not possible)

**Have you spoken to this patient about how cancer might influence their life expectancy?**

- Yes
- No
- No, but I have spoken to their family

**What do you currently estimate the patients’ life expectancy to be?**

- The cancer diagnosis will not affect his/her life expectancy
- More than 5 years
- 2-5 years
- Less than 2 years
- Don’t know this patients’ medical status well enough to tell
- Comorbid illnesses make it difficult to tell

| Please circle the answer that best describes your level of agreement with each item. | Strongly Disagree | Disagree | Agree | Strongly Agree |
| --- | --- | --- | --- | --- |
| **The patient should decide whether we discuss how cancer may affect their life expectancy** | 1 | 2 | 3 | 4 |
| **The cancer doctor should decide whether they discuss with the patient how cancer may affect their life expectancy** | 1 | 2 | 3 | 4 |
| **The patients’ partner/family should decide whether the cancer doctor and patient discuss how cancer may affect their life expectancy** | 1 | 2 | 3 | 4 |

**PSYCHOLOGICAL DISTRESS**

| **What do you estimate the patient’s level of ANXIETY is?** | Normal | Mild | Moderate | Severe |
| --- | --- | --- | --- | --- |
| **What do you estimate the patient’s level of DEPRESSION is?** | Normal | Mild | Moderate | Severe |

***Thank you for taking the time to complete this information***
