# Supplementary material for: Agreement between patients’ and radiation oncologists’ cancer diagnosis and prognosis perceptions: A cross sectional study in Japan
Source: PLoS One. 2018 Jun 8;13(6):e0198437. doi: 10.1371/journal.pone.0198437 (PMC5993258; doi:10.1371/journal.pone.0198437)
Supplement: S3 File — (DOCX) [file pone.0198437.s003.docx]

**S3 File. Clinician survey (Japanese).**

患者さんの癌の種類は何ですか。2種以上のがんが併発している場合は、一番最近に診断された原発性のがんを下記からひとつだけ選んでチェックしてください。

- 乳がん
- 結腸直腸がん
- 前立腺がん
- 肺がん
- 悪性黒色腫
- わかりません
- その他（記述してください）__________________________________

本患者さんはいつ癌と診断されましたか。複数の癌罹患の既往がある場合は最近のものについてお答えください

_____年　_____　月　_____日

放射線治療の主たる目的は何ですか

- がんの治癒
- がん再発の防止
- がんに伴う症状のコントロール（治癒は不可能）

がんが寿命にどのように影響するかについて、この患者に伝えましたか。

- はい
- いいえ
- いいえ、しかし家族とは話した

本患者の現段階での予後についてどのように見積もられますか

- この癌は予後に影響をあたえないと思われる
- 5年より長い
- 2－5年
- 2年未満
- 本患者の病態について十分に把握していない
- 併存疾患のため評価困難

| 本患者について最もあてはまる数字に○をしてください | 全くそう思わない | そう思わない | そう思う | 強くそう思う |
| --- | --- | --- | --- | --- |
| がんが寿命にどのように影響するかの告知について、患者本人が決定するべきである | 1 | 2 | 3 | 4 |
| がんが寿命にどのように影響するかの告知について、主治医が決定するべきである | 1 | 2 | 3 | 4 |
| がんが寿命にどのように影響するかの告知について、患者のパートナー/家族が決定するべきである | 1 | 2 | 3 | 4 |

精神的苦痛について、あてはまる程度に○をしてください

| **本患者の不安の程度はどのくらいだと考えますか** | 正常 | 軽度 | 中等度 | 高度 |
| --- | --- | --- | --- | --- |
| **本患者の抑うつの程度はどのくらいだと考えますか** | 正常 | 軽度 | 中等度 | 高度 |

**以上です**

**ご協力ありがとうございました**
