# Supplementary material for: Agreement between patients’ and radiation oncologists’ cancer diagnosis and prognosis perceptions: A cross sectional study in Japan
Source: PLoS One. 2018 Jun 8;13(6):e0198437. doi: 10.1371/journal.pone.0198437 (PMC5993258; doi:10.1371/journal.pone.0198437)
Supplement: S4 File — (DOC) [file pone.0198437.s004.doc]

**S4 File. STROBE Statement—Checklist of items that should be included in reports of cross-sectional studies.**

|  | Item No | Recommendation |
| --- | --- | --- |
| **Title and abstract** | 1 | (*a*) Indicate the study’s design with a commonly used term in the title or the abstract  “Agreement between patients’ and radiation oncologists’ cancer diagnosis and prognosis perceptions: A cross sectional study in Japan” |
|  |  | (*b*) Provide in the abstract an informative and balanced summary of what was done and what was found  See page 2 |
| Introduction | | |
| Background/rationale | 2 | Explain the scientific background and rationale for the investigation being reported  See page 3, lines 1-24 |
| Objectives | 3 | State specific objectives, including any prespecified hypotheses  See page 3, lines 24-28 |
| Methods | | |
| Study design | 4 | Present key elements of study design early in the paper  See “Study design” section on page 4 |
| Setting | 5 | Describe the setting, locations, and relevant dates, including periods of recruitment, exposure, follow-up, and data collection  See “Sample and Setting” section on page 4 |
| Participants | 6 | (*a*) Give the eligibility criteria, and the sources and methods of selection of participants  See “Sample and Setting” and “Procedure” sections on page 4 |
| Variables | 7 | Clearly define all outcomes, exposures, predictors, potential confounders, and effect modifiers. Give diagnostic criteria, if applicable  See “Measures” section on pages 4-5 and Supplementary Files. |
| Data sources/ measurement | 8* | For each variable of interest, give sources of data and details of methods of assessment (measurement). Describe comparability of assessment methods if there is more than one group  See “Measures” section on pages 4-5 and Supplementary Files. |
| Bias | 9 | Describe any efforts to address potential sources of bias  See “Statistical analysis” section on pages 5-6. |
| Study size | 10 | Explain how the study size was arrived at  See “Sample size” section on page 6. |
| Quantitative variables | 11 | Explain how quantitative variables were handled in the analyses. If applicable, describe which groupings were chosen and why  See “Statistical analysis” section on pages 5-6. |
| Statistical methods | 12 | (*a*) Describe all statistical methods, including those used to control for confounding  See “Statistical analysis” section on pages 5-6. |
|  |  | (*b*) Describe any methods used to examine subgroups and interactions  See “Statistical analysis” section on pages 5-6. |
|  |  | (*c*) Explain how missing data were addressed  See “Statistical analysis” section on pages 5-6. |
|  |  | (*d*) If applicable, describe analytical methods taking account of sampling strategy  N/A |
|  |  | (*e*) Describe any sensitivity analyses  N/A. |
| Results | | |
| Participants | 13* | (a) Report numbers of individuals at each stage of study—eg numbers potentially eligible, examined for eligibility, confirmed eligible, included in the study, completing follow-up, and analysed  See “Consent rates- patient and radiation oncologist survey” pages 6-7. |
|  |  | (b) Give reasons for non-participation at each stage  See “Consent rates- patient and radiation oncologist survey” pages 6-7. |
|  |  | (c) Consider use of a flow diagram  Not included. |
| Descriptive data | 14* | (a) Give characteristics of study participants (eg demographic, clinical, social) and information on exposures and potential confounders  See “Consent rates- patient and radiation oncologist survey” pages 6-7. |
|  |  | (b) Indicate number of participants with missing data for each variable of interest  See Tables 1-4. |
| Outcome data | 15* | Report numbers of outcome events or summary measures  See pages 7-10. |
| Main results | 16 | (*a*) Give unadjusted estimates and, if applicable, confounder-adjusted estimates and their precision (eg, 95% confidence interval). Make clear which confounders were adjusted for and why they were included  See pages 7-10, “Statistical analysis” pages 5-6, Tables 1-4 & Figure 1. |
|  |  | (*b*) Report category boundaries when continuous variables were categorized  See “Statistical analysis” section on pages 5-6 & S4 Table. |
|  |  | (*c*) If relevant, consider translating estimates of relative risk into absolute risk for a meaningful time period  N/A |
| Other analyses | 17 | Report other analyses done—eg analyses of subgroups and interactions, and sensitivity analyses  N/A |
| Discussion | | |
| Key results | 18 | Summarise key results with reference to study objectives  See pages 10-13. |
| Limitations | 19 | Discuss limitations of the study, taking into account sources of potential bias or imprecision. Discuss both direction and magnitude of any potential bias  See page 12, lines 7-14. |
| Interpretation | 20 | Give a cautious overall interpretation of results considering objectives, limitations, multiplicity of analyses, results from similar studies, and other relevant evidence  See pages 10-13. |
| Generalisability | 21 | Discuss the generalisability (external validity) of the study results  See page 12, lines 7-14. |
| Other information | | |
| Funding | 22 | Give the source of funding and the role of the funders for the present study and, if applicable, for the original study on which the present article is based  Information included in online submission form. |

*Give information separately for exposed and unexposed groups.
