# Supplementary material for: Agreement between patients’ and radiation oncologists’ cancer diagnosis and prognosis perceptions: A cross sectional study in Japan
Source: PLoS One. 2018 Jun 8;13(6):e0198437. doi: 10.1371/journal.pone.0198437 (PMC5993258; doi:10.1371/journal.pone.0198437)
Supplement: S1 Table — (DOCX) [file pone.0198437.s005.docx]

**S1 Table. Classification of patients’ and clinicians’ non-mutually exclusive preferences for life expectancy disclosure.**

| **Preference classification:** | ***Patient response:*** | ***Clinician response:*** |
| --- | --- | --- |
| **Preference for patient-determined disclosure of life expectancy information** | Agreed or strongly agreed that they *“would prefer my radiation oncologist to ask me if I want to discuss life expectancy”* | Agreed or strongly agreed that *“the patient should decide whether we discuss how cancer may affect their life expectancy”* |
| **Preference for partner/family-determined disclosure of life expectancy information** | Agreed or strongly agreed that they *“would prefer my radiation oncologist to tell my partner or family and let them decide whether I should be told”* | Agreed or strongly agreed that *“the patients’ partner/family should decide whether the cancer doctor and patient discuss how cancer may affect their life expectancy”* |
| **Preference for clinician-determined disclosure of life expectancy information** | Agreed or strongly agreed that they *“would prefer my radiation oncologist to just tell me the news he/she thinks I can cope with”* | Agreed or strongly agreed that *“the cancer doctor should decide whether they discuss with the patient how cancer may affect their life expectancy”* |
